# Climate of Accountability, Respect, and Ethics Survey (CARES): development and validation of an organizational climate survey

**DOI:** 10.3389/frma.2025.1516726

**Published:** 2025-02-25

**Authors:** Brian C. Martinson, Jarvis Smallfield, Vicki J. Magley, Carol R. Thrush, C. K. Gunsalus

**Affiliations:** ^1^Retired, Saint Paul, MI, United States; ^2^Department of Management, Central Michigan University, Mount Pleasant, MI, United States; ^3^Department of Psychological Sciences, University of Connecticut, Mansfield, CT, United States; ^4^Department of Surgery, University of Arkansas for Medical Sciences, Little Rock, AR, United States; ^5^National Center for Principled Research and Ethics, University of Illinois Urbana-Champaign, Champaign, IL, United States

**Keywords:** organizational climate, workplace civility, harassment, accountability, psychometrics, scale development

## Abstract

**Background:**

This research describes the development and validation of the CARES Climate Survey, a 22-item measure designed to assess interpersonal dimensions of work-unit climates. Dimensions of work-unit climates are identified through work-unit member perceptions and include civility, interpersonal accountability, conflict resolution, and institutional harassment responsiveness.

**Methods:**

Two samples (*N* = 1,384; *N* = 868) of academic researchers, including one from the North American membership of the American Geophysical Union (AGU), and one from a large research-intensive university, responded to the CARES and additional measures via an online survey.

**Results:**

We demonstrate content validity of the CARES measure and confirm structural validity through exploratory and confirmatory factor analyses which yielded four dimensions of interpersonal climate. In addition, we confirm the CARES internal reliability, construct validity, and excellent sub-group invariance.

**Conclusions:**

The CARES is a brief, psychometrically sound instrument that can be used by researchers, institutional leaders, and other practitioners to assess interpersonal climates in organizational work-units.

**Originality/value:**

This is the first study to develop and validate such a measure of interpersonal climates specifically in research-intensive organizations, using rigorous psychometric methods, grounded in both theory and prior research on work-unit climates.

## Introduction

Organizational members who are skilled and competent in work-related tasks is a necessary, but not sufficient condition for a highly functioning organization. Creating and maintaining the highest levels of organizational effectiveness, productivity, and integrity requires members' full engagement with their work. To support full engagement, organizational members must experience psychological safety, feel respected and valued, and have a sense of positive interpersonal interactions (Bryer, 2020; Frazier et al., 2017; Knapp et al., 2014; Komisarof, 2022). Because most organizational members must interact with others to get their work done, the nature, competence, and integrity of their interpersonal interactions influences the quality and quantity of work performed as well as relevant psychological outcomes for the members themselves (Liden et al., 2000; Reich and Hershcovis, 2011). Organizational cultures and climates have been identified as important facilitators or modifiers of interpersonal interactions, both positively and negatively (Andersson and Pearson, 1999; Willness et al., 2007) and correlate with the integrity of creative efforts such as research in academic settings; with positive climates being associated with higher integrity behavior and negative climates being associated with lower integrity behavior (Crain et al., 2013). Work units that provide climates of respect and integrity also support the best possible outcomes for individual organizational members (Feldblum and Lipnic, 2016; Frone, 2000; NASEM, 2018), their performance (Carr et al., 2003), and their creativity (Tu et al., 2019). Thus, interpersonal climates in organizations are key to the success of its members, and the ability to measure such climates can be a powerful strategy for minimizing negative interpersonal interactions and maximizing their quality and integrity (Walsh and Magley, 2019).

Effective leadership in organizations must include a focus on interpersonal aspects of the workplace and psychologically safe workplaces (Cross et al., 2019; Haven et al., 2021; Valantine et al., 2021). A primary goal of organizational administration and leadership is to facilitate members' ability to produce their best work. Yet, the nature of large institutions can leave leaders somewhat removed from the day-to-day interactions in the organizational sub-units over which they have charge. Without valid and useful information about the interactions in units across their organizations, how can these leaders know how well their organizational settings or climates support their members and foster the highest integrity of work with effectiveness, accountability, and respect? The purpose of our work here is to develop and validate a psychometrically sound survey to assess interpersonal work-unit climates, particularly within but not limited to academic research-focused environments. Members of our team have long been measuring research integrity climate and civility in organizations (Martinson et al., 2013; Walsh et al., 2011) but realized the lack of validated tools for assessing micro-climates relevant to lab/team level interpersonal dimensions that are important to maintaining accountable, ethical, respectful working environments.

There are two fairly distinct categories of prior work on organizational climate, one that has emphasized global or generic climate, and a second that has emphasized a strategic focus on specific aspects of organizational climates such as service, safety, justice, diversity, civility, and integrity. While multiple of these foci overlap to some extent with interpersonal climate, we have not identified any climate instrument that adequately captures interpersonal climate, despite the fact that many climate measures contain large numbers of scale items. Many existing climate assessment tools also either downplay or entirely ignore the role of power and power-differentials in organizations as factors that affect organizational member behavior and outcomes. Yet, in academic research environments, supervision dynamics, particularly inefficient supervision has been identified as an important barrier to fostering responsible research climates (Haven et al., 2020; Kis et al., 2022). Moreover, in much prior work the voices of women and those of various under-represented groups are not much represented. The development of the climate measure described here has been informed by a comprehensive understanding of interpersonal climate and an effort to understand how that climate is manifest among members of workgroups in organizations.

Contextual factors, what we refer to as organizational climate, have been identified as precursors to problematic interpersonal behavior (Andersson and Pearson, 1999; Pearson et al., 2000). Time-pressures, resource uncertainties, and strong status hierarchies can create opportunities for disrespectful interpersonal interactions between organizational members that over time can become entrenched as workplace norms (Pearson et al., 2001), in turn, leading to disengagement, disaffection, and behaviors that run counter to desired organizational outcomes (Pearson and Porath, 2005).

Among many problematic interpersonal behaviors, sexual harassment has perhaps received the greatest attention and has been the focus of perhaps the most organizational research and policy (NASEM, 2018). It is also a type of behavior in organizations for which laws exist as sanctions against its occurrence. Importantly, sexual harassment has also been contextualized in a way that suggests it is better understood not primarily as sexualized behavior, but as a misuse and abuse of power, that often takes sex-based forms. In their study of 8th Circuit Federal Court employees, Lim and Cortina (2005) document significant correlations between sexualized harassment and incivility, and even stronger correlations between incivility and the construct of gender harassment, the most frequent sub-type of sexualized harassment which reflects sex-related put-downs and sexism. Others have widened the window of discourse to include toxic leadership, bullying, and harassment, and to distinguish behaviors oriented toward expressing dominance over others from strictly sexually oriented behavior (Berdahl et al., 2018; Berdahl and Bhattacharyya, 2021; Glick et al., 2018). Many of these authors have noted that disrespectful, hostile, and intimidating behavior may be targeted toward numerous groups of organizational members based on multiple dimensions of their identities, often intersectionally.

While organizational climates have often been demonstrated to contribute to many types of *undesirable* interpersonal behavior, efforts to develop more *positive* organizational climates can help sustain prosocial norms in the workplace that promote members' sense of safety, respect, and belonging (Feldblum and Lipnic, 2016; NASEM, 2018; Walsh and Magley, 2019). Interpersonal collaboration, itself predicated on genuine respect between co-workers, has been shown to be more important than a sense of purpose in maximizing employee engagement (Cross et al., 2019). Moreover, the quality of interpersonal aspects of work have also been identified as a key component of worker wellbeing (Lovejoy et al., 2021). Our work here is concerned with the kinds of detrimental interpersonal behaviors that have previously been characterized by Andersson and Pearson (1999) as “low-intensity deviant behavior with ambiguous intent to harm the target, in violation of workplace norms for mutual respect” (p. 457).

Concerns about interpersonal misbehavior in research organizations is directly connected to, though not limited to, concerns about research integrity. In recent years, the mistreatment of individuals (e.g., sexual harassment), has been increasingly recognized as undermining excellence in research, and being a form of research misconduct in and of itself. Examples of this include the 2018 NASEM Report on Harassment in STEMM (NASEM, 2018), the inclusion of “discrimination, harassment (including sexual harassment), and bullying” as forms of research misconduct in the American Geophysical Union (AGU)'s 2017 Integrity and Ethics Policy in its Code of Conduct (https://www.agu.org/-/media/Files/Learn-About-AGU/AGU_Scientific_Integrity_and_Professional_Ethics_Policy_document.pdf), the September 2018 announcement by the NSF of new reporting requirements by institutions of sexual harassment on the part of NSF research grantees (Mervis and Kaiser, 2018), and a subsequent, comparable strengthening of polices toward sexual harassment on the part of NIH (Kaiser, 2019). Given the potential importance of organizational climate, it is unfortunate that interpersonal organizational climate has generally received less attention than other organizational factors that influence the quality of outputs and members' wellbeing. Based on our review of existing measures, there has not been a comprehensive instrument proposed or developed to assess the broader scope of interpersonal organizational climates with which we are concerned. Until now, while specific measures of incivility climate have been developed— Civility Norms Questionnaire-Brief (CNQ-B) (Walsh et al., 2011) and the Survey of Organizational Research Climates (SOURCE), for assessing research integrity climates in academic research settings (Martinson et al., 2013)— we have found no instrument designed specifically to assess climates of organizational work-units that includes the constellation of civility, respect, misuses of power (e.g., harassment, bullying), and conflict resolution. It should be noted that instruments exist for assessing sexual assault and harassment in academic settings (Cantor et al., 2015), but these fall short of the goal of broadly assessing interpersonal climate. On the one hand, extant instruments fail to assess organizational climates, rather querying direct personal experiences, and on the other hand, lack complete coverage of important dimensions of these climates. To be clear, we did not seek to develop a measure of the frequency or prevalence of interpersonal interactions whether respectful or harassing. We have specifically avoided inclusion of experientially framed survey items that tap concepts such as harassment frequency.

In defining organizational climate, we follow the definition offered by Ehrhart, Schneider and Macey that it is “the shared meaning organizational members attach to the events, policies, practices, and procedures they experience and the behaviors they see being rewarded, supported, and expected” (Ehrhart et al., 2013, p. 115). The factors identified by Ehrhart et al. as markers of organizational climate are the observable (and therefore reportable) aspects of organizational life that point to the deeper organizational culture. Thus, we sought to develop a measure with items that focus on such observable and reportable features of members' working environments. Emergent dimensions of interpersonal climate addressed by these items include a broad spectrum from the positive (psychological safety and civility) to the negative (bullying, harassment, assault).

Although not encompassing of a broad understanding of interpersonal climate, we expect the psychological safety experienced by individuals, and the protection of a sense of psychological safety through accountability for misbehavior, respectful conflict resolution, and institutional level supports for positive interpersonal interactions, are formative of a positive interpersonal workgroup climate. Psychological safety is considered present when members hold perceptions that they are able to engage in interpersonal risk taking within the workgroup without fear of negative personal or professional consequences (Edmondson, 1999; Kahn, 1990) and is instrumental in the willingness to share ideas and act in concert with others (Frazier et al., 2017). Critical aspects to the perceptions of psychological safety include a sense of support and respect from other workgroup members, the creation of expectations and norms through leader behavior, and the expectation that institutions place importance on these group norms and interpersonal relationship expectations (Frazier et al., 2017). Therefore, we sought to develop a measure that includes items reflecting all three perceptual levels of contribution to interpersonal climates—co-workgroup member interaction climate, leader influence, and institutional practices and processes.

Finally, it is important to note that workgroup-level interpersonal climate as conceptualized here is a necessarily broad concept which encompasses relevant theoretical aspects and levels of influence, because members do not cognitively map all potential aspects nor hold each with the same level of salience. In order to create a maximally useful and applicable measure, capturing the totality of interpersonal climates, and that is sufficiently brief to be employable in most organizational research studies, we engaged in a predominately inductive process that captured a comprehensive representation of interpersonal organizational climate. In this article we describe the development of this new measure and evaluate its psychometric properties including content validity, structural validity internal reliability, as well as construct validity and factorial test invariance for multiple characteristics of respondent subgroups (i.e., race, gender, career stage).

## Method and results

### Scale development overview

**Figure 1:**
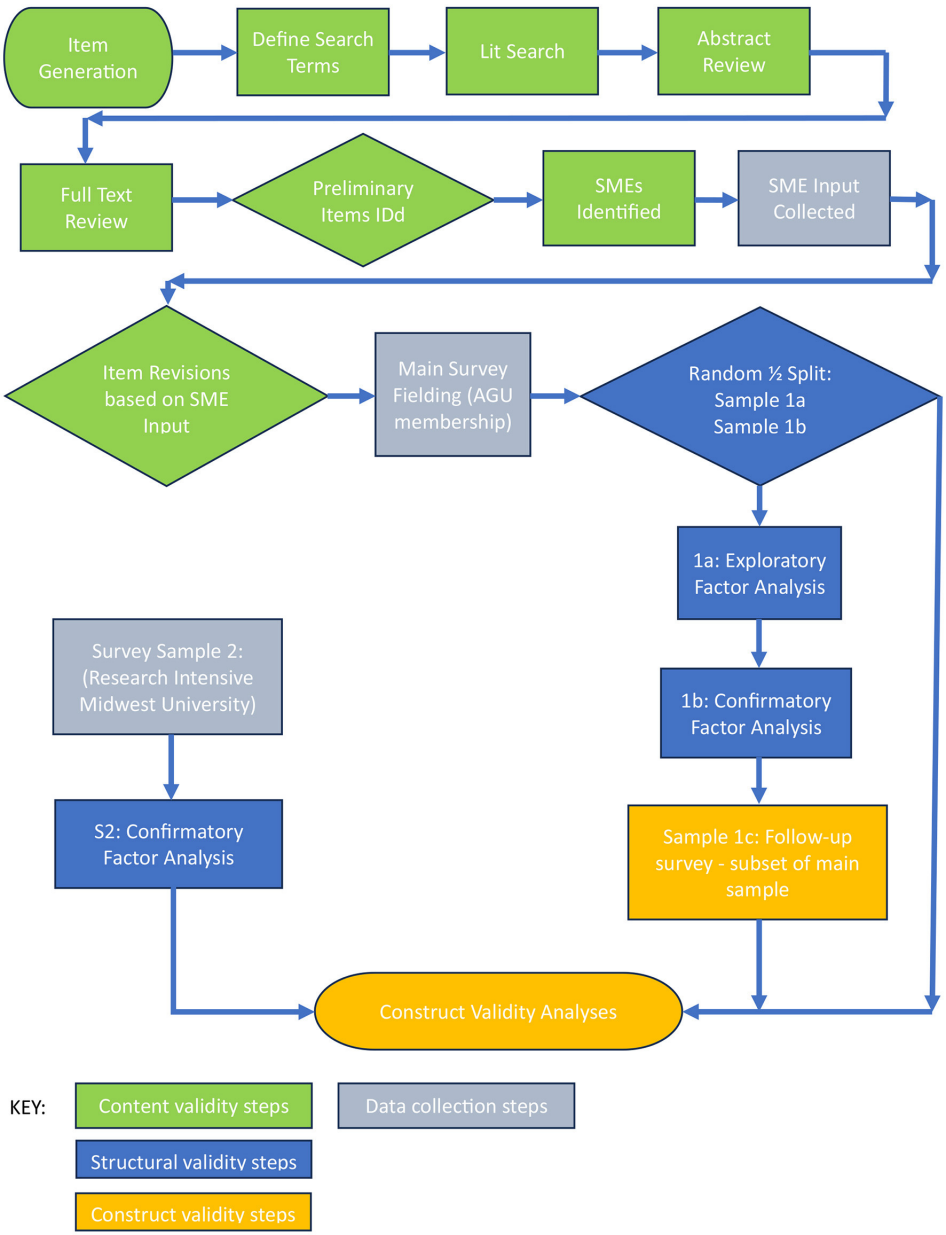
CARES development and validation flowchart.

In developing the interpersonal climate survey we relied on broadly accepted scale development methods (e.g., Hinkin, 1998; Rahim and Magner, 1995) across multiple phases, including literature review, item generation, content validation, exploratory and confirmatory factor analyses, and nomological validity estimates. This process produced a 22-item scale and revealed four distinct dimensions of the scale. It should be noted that while we believe this measure which we named the Climate of Accountability, Respect and Ethics Survey (CARES) is suitable for use in a broad range of organizational settings, the empirical work on which we report here was conducted primarily in research settings, including, but not limited to universities. Please refer to the flowchart in Figure 1 for a map of our work-flow.

### Item generation

We worked with a professional librarian to conduct a search of online databases including PubMed, Sociological Abstracts, Web of Science, Academic Search Premier, and Business Source Premier for existing scales and surveys covering our conceptual dimensions of interest using specific search-terms including harassment, assault, bullying, intimidation, psychological safety, civility, and organizational climate. We combined those results with additional manuscripts identified by team members and other content experts resulting in a final collection of 198 articles, manuscripts, and reports. All abstracts and summaries of these materials were reviewed by three team members (BCM, JS, CRT) who identified 45 sources as relevant for a full-text review. This review resulted in an initial pool of 618 scale items covering a breadth of conceptual dimensions (including psychological safety, civility, bullying, harassment, and assault) across the domains of policies, procedures, leadership practices, socialization, norms, expectations, and institutional resources. Those items were subjected to an iterative two-rater process to sort and organize each by dimension and domain. We culled items with substantially duplicate content and evaluated the remaining items to ensure topic coverage, reducing the pool to 132 items covering all conceptual domains and which were not substantially duplicative. See Supplementary materials 2, 3 files for further description of our process here, and the artifacts of that process.

This pool of 132 items provided a conceptual landscape of domains of focus for existing scales. Four-members of our research team (BCM, JS, VM, CRT) extensively reviewed this pool of items, discussing as a team to reach consensus to further reduce the number of candidate items by culling experiential and poorly worded items, recrafting experientially worded items into climate-focused items, creating new items where concepts were found to be missing, and reframing item wording as necessary. This final stage of initial item generation produced 61 items for further consideration addressing climate across all conceptual domains.

#### Subject matter expert review

The authors invited 35 subject matter experts (SME's) to review and provide feedback on the 61 candidate items. The invited pool consisted of 23 women and 12 members of minority racial groups. SME's were chosen based on one or more of the following criteria: (1) expert scholars in organizational psychology, sexual misconduct, harassment, psychological safety, or professionalism in academia, (2) panel members of the 2018 NASEM Report on Sexual Harassment (NASEM, 2018), (3) panel members of the 2019 NIH Working Group report on sexual harassment (Kaiser, 2019), (4) academic professionals (e.g., journal editors) with experience and interest in interpersonal working conditions, and (5) graduate students or postdoctoral research fellows. Of the invited pool, 18 SME's provided quantitative ratings indicating the extent to which each item was relevant to workplace climate as well as extensive qualitative feedback. See Supplemental material 1 for further details about the SME identification and recruitment process, as well as details regarding the characteristics of the SME candidate pool and ultimate participants, what input we requested from them and how that was processed, as well as the instructions and data-collection forms provided to SMEs. The authors engaged in extensive analysis and discussion of these results using a consensus approach that led to substantial revisions to most items and a further vetting process which produced a final list of 46 items which we carried forward for large-scale data collection and psychometric analysis.

#### Sample 1 factor analysis participants

We invited individuals from the North American membership of the American Geophysical Union (AGU), an international nonprofit scientific professional association of earth and space scientists, who were active researchers to complete online surveys designed to assess their work-unit climates. See Supplemental material 4 for a copy of the survey instrument as it was implemented in Qualtrics. Participation was voluntary and participants were assured of the confidentiality of the study. All study activities reported in this paper were approved by the University of Illinois Urbana-Champaign Institutional Review Board. A total of 27,753 members were invited, and among 2,572 who consented to participate (9%), 2,253 reported being actively engaged in research and therefore eligible to participate (87% of those consenting). We omitted a further 869 due to suspect or missing responses, ultimately resulting in 1,384 usable surveys, representing 61% of the eligible, consenting participants. Responses were considered missing if half or more of the climate response items remained unanswered. Responses were considered suspect if all items in a climate scale were answered with the same response value. The demographic breakdown was as follows: 45% female, 50% male, and 4.6% not answering; 0.2% Native Hawaiian or Pacific Islander, 0.3% American Indian or Alaskan Native, 1% Black or African American, 5% Hispanic or Latino, 8% Asian, 73% white, 6% not listed or multiracial, and 6% not answering; 16% students, 29% early career researchers, 27% mid-career researchers, 23% late career researchers, and 21% not answering. We divided the Sample 1 data set into two random samples of 692 individuals each (Sample 1a and Sample 1b in Figure 1) to conduct exploratory factor analysis (EFA) and confirmatory factor analysis (CFA). When evaluating model fit for the purposes of factor analysis, the Comparative Fit Index (CFI), Tucker-Lewis Index (TLI), root mean squared error of approximation (RMSEA), and standardized root mean squared residual (SRMR) of the models were considered. Cutoff values of 0.95 for CFI and TLI, 0.06 for RMSEA, and 0.08 for SRMR indices were used as indications of relatively good fit (Hu and Bentler, 1999). All analyses were conducted using R version 4.0.3 utilizing maximum likelihood (ML) estimation unless otherwise noted.

#### Measures

Response options for all candidate items were scaled from 1 (Not at All) to 5 (Completely) with an additional “No Basis for Judging” option. We modeled the response scales here from those we previously employed in the SOURCE (Martinson et al., 2013). Scale reliabilities for the CARES dimensions and for the overall scale are reported for the CFA sample.

##### Workgroup climate (CARES)

The interpersonal climate of the workgroup was measured with the 22-item CARES climate scale developed as described below and items for which are found in Appendix 1.

##### Research integrity climate (SOURCE)

Participants rated the research integrity climate of their workgroup and institution with the 28-item SOURCE climate scale from Martinson et al. (2013). An example item from the SOURCE is “How true is it that pressure to publish has a negative effect on the integrity of research in your department?” (α = 0.95, ω = 0.96).

##### Integration

Participants rated their feeling of being integrated into their department with a single-item, “How integrated within your department do you feel?”

##### Continuance commitment

Participants rated their level of continuance commitment to their organization using an abridged 3-item version of the continuance scale from Allen and Meyer (1990). An example item is “It would be very hard for me to leave my organization right now, even if I wanted to (α = 0.78, ω = 0.78).

#### Exploratory factor analysis—sample 1a

We subjected items resulting from the subject matter expert review portion of development to an EFA using principal components analysis with an unspecified number of factors and utilizing the first split half of Sample 1 (Sample 1a in Figure 1). Four factors with eigenvalues >1 were revealed. These results were supported by a parallel analysis of an uncorrelated matrix representing random sampling error where factors generated by the EFA with eigenvalues greater than the corresponding eigenvalue from the parallel matrix represent underlying components. The results were further supported by an optimal coordinates analysis where factors with eigenvalues which diverge from (i.e., exceed) a regression line from the last eigenvalue through the preceding eigenvalue also returned four factors. Finally, we verified these results through visual inspection of the scree plot.

Because of anticipated intercorrelation among factors, we employed oblique rotation to interpret loadings of the items onto each factor. We interpreted each factor based on items which loaded onto that factor with weights above 0.5 and with no cross loading of more than 0.3 onto other factors. To create a reliable scale, we eliminated items with overly ambiguous loading patterns or that loaded poorly on their primary factor. We identified the CARES scale as one construct good internal reliability (α = 0.95, ω = 0.96) consisting of four dimensions identified after rotation and consisting of the following number of items: civility climate (a climate of respectful and supportive treatment of other workgroup members consisting of 7 items with internal reliability α = 0.90, ω = 0.92), interpersonal accountability climate (a climate where hostile, intimidating, and unhealthy interpersonal behaviors are not tolerated consisting of 7 items with internal reliability α = 0.93, ω = 0.94), conflict resolution climate (a climate where leaders expect and facilitate respectful resolution of interpersonal conflict consisting of 3 items with internal reliability α = 0.71, ω = 0.76), and institutional harassment responsiveness (institutional level policies and behaviors which unambiguously communicate that the institution does not tolerate harassment consisting of 5 items with internal reliability α = 0.94, ω = 0.94).

#### Confirmatory factor analysis—sample 1b

The next step in validating the 22-item workgroup climate scale was to perform a CFA on the second split half of Sample 1, which produces assessments of goodness of fit and can be used to confirm previously hypothesized models. The CFA was based on the covariance matrix and used maximum likelihood estimation, and the results are shown in Table 1.

**Table 1:** Sample 1b confirmatory factor analyses (second split half).

**Model**	**df**	**χ2**	**Δ χ2**	**CFI**	**TLI**	**RMSEA**	**SRMR**
4-factor expected	203	747.281	10,832.885	0.95	0.95	0.062	0.043
3-factor^a^	206	1,070.503	10,509.663	0.92	0.92	0.078	0.054
3-factor^b^	206	1,615.262	9,964.904	0.88	0.86	0.099	0.063
2-factor^c^	208	2,001.781	9,578.385	0.84	0.82	0.112	0.072
1-factor^d^	209	4,552.028	7,028.138	0.62	0.58	0.173	0.128

*N* = 692, ^a^civility and conflict resolution dimensions combined, ^b^civility and interpersonal accountability climate dimensions combined, ^c^civility, interpersonal accountability, and conflict resolution dimensions combined, ^d^all dimensions combined.

Consistent with our EFA results, our hypothesized model was a 4-factor model with seven items each for the civility and interpersonal accountability climate dimensions, three items for the conflict resolution climate dimensions, and five items for the institutional harassment responsiveness dimension. The hypothesized model produced a chi-squared value of 747.281 (df = 203, *p* < 0.01), and the fit indices indicated a good overall fit (CFI = 0.95; RMSEA = 0.062; SRMR = 0.043). We tested several alternative models, and all failed to produce a better fit than the four-factor model. Thus, the hypothesized model derived from the EFA was confirmed. The means, standard deviations, and correlations for the second split half of Sample 1 can be found in Table 2.^1^

**Table 2:** Sample 1 means, standard deviations, and correlations among variables.

	**Mean**	**SD**	**1**	**2**	**3**	**4**	**5**	**6**	**7**	**8**	**9**	**10**
1. CARES^a^	3.17	0.78										
2. Civility^a^	3.72	0.89	0.84^***^									
3. Accountability^a^	3.08	0.91	0.79^***^	0.73^***^								
4. Conflict^a^	2.73	1.03	0.80^***^	0.59^***^	0.44^***^							
5. Responsiveness^a^	3.27	1.15	0.75^***^	0.42^***^	0.39^***^	0.48^***^						
6. SOURCE^a^	3.55	0.67	0.78^***^	0.79^***^	0.60^***^	0.54^***^	0.56^***^					
7. Integration^a^	3.33	1.20	0.55^***^	0.51^***^	0.47^***^	0.35^***^	0.37^***^	0.60^***^				
8. Continuance^a^	3.20	1.04	−0.20^***^	−0.18^***^	−0.23^***^	−0.15^***^	−0.11^**^	−0.27^***^	−0.11^**^			
9. OCB^b^	3.82	0.90	0.58^***^	0.60^***^	0.53^***^	0.48^***^	0.21^**^	0.55^***^	0.40^***^	−0.09		
10. WUD^b^	3.99	0.94	0.72^***^	0.76^***^	0.68^***^	0.47^***^	0.35^***^	0.63^***^	0.45^***^	−0.12	0.71^***^	
11. WUE^b^	3.87	0.73	0.66^***^	0.73^***^	0.69^***^	0.54^***^	0.26^**^	0.57^***^	0.34^***^	−0.16^*^	0.66^***^	0.76^***^

Integration = feelings of being integrated into the workgroup, Continuance = continuance commitment, OCB = organizational citizenship behaviors, WUD = work unit diversity climate, WUE = work unit experiences, ^a^time 1 constructs, *N* = 692, ^b^time 2 constructs, *N* = 343, ^*^p < 0.05, ^**^p < 0.01, ^***^p < 0.001. *P*-values < = 0.05 indicate that the observed/reported value in the table is statistically significantly different from zero applying the typical probability threshold of 0.05. *P*-values smaller than that indicate the same statistical significance, but at increasingly stringent probability thresholds.

Members of workgroups may perceive working environments differently based on individual identity, group membership, career rank, and other factors. It is therefore instructive to test the current instrument for measurement invariance across groups to ensure that members of different groups perceive scale items similarly. Evaluating measurement invariance across groups aids in establishing the efficacy of the instrument to investigate differences in perception across groups in addition to perceptions of workgroup members in general. We conducted multigroup confirmatory factor analysis to test the invariance of parameters of nested models across groups where subsequent sets of parameters are allowed to vary across groups in one model and constrained to be constant across groups in the nested model. If model fit is similar in both the unconstrained and constrained models, we can conclude that the model is invariant across groups for the targeted parameter. We evaluated the CARES instrument sequentially across four levels of invariance: configural invariance (indicating that the model contains the same number of factors with the same items loading onto each factor across groups), metric invariance (indicating that item factor loadings are equivalent across groups), scalar invariance (indicating that slope intercepts are equivalent across groups), and strict invariance (indicating that the error variance is equivalent across groups). Achieving scalar invariance indicates that both the model structure and observed mean scores are comparable across groups (Steenkamp and Baumgartner, 1998; Steinmetz, 2013). Strict invariance is unnecessary to establish meaningful measurement comparisons across groups (Luong and Flake, 2023; Steenkamp and Baumgartner, 1998), however, we include those results here for interest and completeness.

We tested invariance across several groups. First, we examined invariance for those who reported themselves as belonging to either the majority or minority gender in their workgroup, and the majority or minority race in their workgroup. We had enough participants to further examine invariance across the four intersections of these categories. We further examined the scale dependent on whether participants reported their sex as female or male. We did not have enough of each category to examine invariance across all reported racial groups. We grouped responses into the racial groups of BIPOC and non-BIPOC, which provided sufficient responses in each group to examine scale invariance across groups. Finally, we examined scale invariance across individuals who reported themselves as being in four different career stages (student, early career, mid-career, and experienced). Measurement invariance is confirmed when ΔCFI < 0.010 between nested models (Cheung and Rensvold, 2002). Results are presented in Table 3 and demonstrate the strongly consistent performance of the scale across groups, showing configural, metric, and scalar invariance across all groups and intersections of groups of interest collected in this sample and strict invariance across most of those groups. These results provide substantial confidence in the use of the CARES scale to meaningfully interpret and compare perceptions of participants from many different groups.

**Table 3:** Sample 1b multi-group confirmatory factor analyses (second split half).

**Group**	**Level**	**df**	**χ2**	**RMSEA**	**SRMR**	**CFI**	**Δ CFI**
Majority/minority gender^a^	Configural	406	997.853	0.066	0.049	0.946	
	Metric	424	1,027.436	0.065	0.054	0.945	0.001
	Scalar	442	1,055.297	0.064	0.055	0.944	0.001
	Strict	464	1,088.997	0.063	0.055	0.943	0.001
Majority/minority race^b^	Configural	406	1,019.046	0.067	0.050	0.945	
	Metric	424	1,058.276	0.067	0.057	0.943	0.002
	Scalar	442	1,094.657	0.066	0.058	0.941	0.002
	Strict	464	1,168.851	0.067	0.058	0.937	0.004
Majority/minority gender x majority/minority race	Configural	812	1,586.697	0.075	0.057	0.932	
	Metric	866	1,690.449	0.075	0.073	0.927	0.005
	Scalar	920	1,773.856	0.074	0.074	0.925	0.002
	Strict	986	1,972.954	0.077	0.074	0.913	0.012
Sex^c^	Configural	406	978.266	0.065	0.051	0.947	
	Metric	424	1,012.025	0.065	0.057	0.945	0.002
	Scalar	442	1,075.017	0.066	0.058	0.941	0.004
	Strict	464	1,213.795	0.070	0.060	0.930	0.011
Race^d^	Configural	406	1,023.045	0.067	0.049	0.945	
	Metric	424	1,051.120	0.066	0.052	0.944	0.001
	Scalar	442	1,100.477	0.067	0.053	0.942	0.002
	Strict	464	1,169.633	0.067	0.053	0.937	0.005
Sex x race	Configural	812	1,582.385	0.077	0.057	0.930	
	Metric	866	1,689.772	0.077	0.073	0.925	0.005
	Scalar	920	1,826.251	0.078	0.075	0.917	0.008
	Strict	986	2,078.211	0.083	0.077	0.900	0.017
Career stage^e^	Configural	812	1,614.980	0.078	0.059	0.927	
	Metric	866	1,709.711	0.077	0.074	0.923	0.004
	Scalar	920	1,805.140	0.077	0.075	0.919	0.004
	Strict	986	2,106.920	0.084	0.079	0.898	0.021

*N* = 692, ^a^majority or minority gender in the workgroup, ^b^majority or minority race in the workgroup, ^c^female or male, ^d^BIPOC or non-BIPOC, ^e^student, early career, mid-career, or experienced.

The scale showed good configural, metric, and scalar invariance of fit across all respondent groups. Together, these findings support a multidimensional conceptualization of the CARES climate scale, which is durable across member groups, allowing for reliable comparison across groups. Given these findings, we proceeded to explore the ability of workgroup climate to account for variance in group member outcomes (i.e., workgroup integration, organizational citizenship behavior, work unit diversity climate, work unit experiences, continuance commitment to one's employer).

### Sample 2 confirmatory factor analysis

#### Participants

We invited 5,489 individuals from a large, R1, public university in the Midwest of the United States. Our primary purpose for this was to conduct confirmatory factor analyses in a sample of researchers that was more comprehensive of scientific disciplines not represented in the AGU membership sample (Sample 1). Participation was voluntary and participants were assured of the confidentiality of the study. A total of 1,302 consented and reported on a screening item that they were involved in research activities in the university. Similar to sample 1, of these, 549 were omitted due to suspect or missing responses, resulting in a usable sample of 868 participants (a 16% participation rate). The demographic breakdown was as follows: 39% female, 55% male, and 6% not answering; 0.1% American Indian or Alaskan Native, 21% Asian, 3% Black or African American, 6% Hispanic or Latino, 0% native Hawaiian or Pacific Islander, 59% white, 5% not listed or multiracial, and 12% not answering; 34% graduate students, 18% research support including post-doctoral researchers, 41% tenure track faculty, and 8% not answering; 49% early career, 32% mid-career, 18% late career, and 1% not answering.

#### Measures

Scale reliabilities for the CARES dimensions and for the overall scale are reported for the CFA sample.

#### Workgroup climate (CARES)

The interpersonal climate of the workgroup was measured with the 22-item CARES climate scale developed here (α = 0.94, ω = 0.97). The reliabilities of the dimension of the scale were: civility climate (α = 0.90, ω = 0.93), interpersonal accountability climate (α = 0.94, ω = 0.96), conflict resolution climate (α = 0.79, ω = 0.84), and institutional harassment responsiveness (α = 0.95, ω = 0.96).

#### Research integrity climate (SOURCE)

Participants rated the research integrity climate of their workgroup and institution with the 28-item SOURCE climate scale (Martinson et al., 2013) (α = 0.94, ω = 0.95).

### Sample 2 results

#### Confirmatory factor analysis

As with Sample 1b, we validated the 22-item workgroup climate scale by conducting a CFA against the data obtained in this sample with the results reported in Table 4. The hypothesized model produced a chi-square value of 753.356 (df = 203, *p* < 0.01), and the fit indices indicated a good overall fit (CFI = 0.97; RMSEA = 0.056; SRMR = 0.034). We tested several alternative models, and all failed to produce a better fit than the four-factor model. Thus, the hypothesized model obtained with Sample 1b was confirmed.

**Table 4:** Sample 2 confirmatory factor analyses.

**Model**	**df**	**χ2**	**Δ χ2**	**CFI**	**TLI**	**RMSEA**	**SRMR**
4-factor expected	203	753.356	15,521.342	0.97	0.96	0.056	0.034
3-factor^a^	206	1,758.711	14,515.987	0.90	0.89	0.093	0.048
3-factor^b^	206	2,630.571	13,644.127	0.85	0.83	0.116	0.093
2-factor^c^	208	3,931.055	12,343.643	0.77	0.74	0.144	0.098
1-factor^d^	209	7,747.842	8,526.856	0.53	0.48	0.204	0.151

*N* = 868, ^a^civility and conflict resolution dimensions combined, ^b^civility and interpersonal accountability climate dimensions combined, ^c^civility, interpersonal accountability, and conflict resolution dimensions combined, ^d^all dimensions combined.

We proceeded to evaluate the CARES instrument sequentially at the four levels of invariance outlined previously across the same breakdown of subgroups as we did with Sample 1b. for several groups available for analysis in this sample. Measurement invariance was confirmed when ΔCFI < 0.010 between nested models (Cheung and Rensvold, 2002). Results are presented in Table 5 and demonstrate the strongly consistent performance of the scale across groups, showing configural, metric, and scalar invariance across all groups and intersections of groups of interest collected in this sample and strict invariance across most of those groups. As with Sample 1b, these results provide substantial confidence in the use of the CARES instrument to meaningfully interpret and compare perceptions of participants from many different groups.

**Table 5:** Sample 2 multi-group confirmatory factor analyses.

**Group**	**Level**	**df**	**χ2**	**RMSEA**	**SRMR**	**CFI**	**Δ CFI**
Majority/minority gender^a^	Configural	406	1,073.345	0.062	0.038	0.959	
	Metric	424	1,084.285	0.060	0.041	0.959	0.000
	Scalar	442	1,110.374	0.059	0.041	0.959	0.000
	Strict	464	1,162.874	0.059	0.042	0.957	0.002
Majority/minority race^b^	Configural	406	1,011.793	0.059	0.039	0.962	
	Metric	424	1,064.510	0.059	0.045	0.960	0.002
	Scalar	442	1,086.384	0.058	0.046	0.960	0.000
	Strict	464	1,170.805	0.059	0.045	0.956	0.004
Majority/minority gender x majority/minority race	Configural	812	1,716.744	0.072	0.047	0.945	
	Metric	866	1,806.015	0.071	0.058	0.943	0.002
	Scalar	920	1,875.215	0.069	0.059	0.942	0.001
	Strict	986	2,091.760	0.072	0.059	0.933	0.009
Sex^c^	Configural	406	946.207	0.057	0.037	0.963	
	Metric	424	987.738	0.057	0.048	0.961	0.001
	Scalar	442	1,019.014	0.056	0.048	0.960	0.001
	Strict	464	1,086.271	0.057	0.048	0.957	0.003
Race^d^	Configural	406	955.818	0.058	0.040	0.962	
	Metric	424	1,007.660	0.059	0.047	0.960	0.002
	Scalar	442	1,067.236	0.059	0.048	0.957	0.003
	Strict	464	1,325.459	0.068	0.049	0.941	0.016
Sex x race	Configural	812	1,455.852	0.063	0.048	0.954	
	Metric	866	1,580.466	0.065	0.066	0.949	0.005
	Scalar	920	1,686.721	0.065	0.067	0.945	0.004
	Strict	986	2,078.354	0.075	0.069	0.922	0.023
Role^e^	Configural	609	1,379.288	0.069	0.049	0.949	
	Metric	645	1,504.282	0.071	0.065	0.943	0.006
	Scalar	681	1,608.371	0.071	0.067	0.938	0.005
	Strict	725	1,864.673	0.077	0.069	0.924	0.014
Years in field^f^	Configural	609	1,317.973	0.064	0.046	0.956	
	Metric	645	1,404.560	0.064	0.057	0.953	0.003
	Scalar	681	1,490.705	0.064	0.058	0.950	0.003
	Strict	725	1,711.215	0.069	0.059	0.939	0.011

*N* = 868, ^a^majority or minority gender in the workgroup, ^b^majority or minority race in the workgroup, ^c^female or male, ^d^BIPOC or non-BIPOC, ^e^graduate student, research support including post-doctoral researchers, or tenure track faculty, ^f^early career (1–5 years), mid-career (6–15 years), or late career (>15 years).

Because of the multi-level nature of influences on workgroup climates, we sought to evaluate the potential for aggregation of scale scores at the group level. Given that the data from Sample 2 was secondary, we did not have the ability to fully specify the information collected. Even though the data did not contain sufficient information to analyze grouping at the workgroup level (the level for which we expect the largest group-level effect for a climate measure), we were able to analyze the data at a higher, unit level of analysis where each unit contained several workgroups. Even though analysis at this level is less than ideal and is expected to result in an underestimation of group-level effects, we proceeded to calculate ICC(1), ICC(2) (Bliese, 2000),


$$ICC\left(1\right),\;\rho =\frac{\sigma_{u_{0}}^{2}}{\sigma_{u_{0}}^{2}+\sigma_{r}^{2}};\;ICC(2),\lambda_{j}=\;\frac{\eta_{j}\rho }{1+(\eta_{j}-\;1)\rho }$$

and r_wg_(j) (James et al., 1993) values for the overall CARES scale and each dimension. The results are found in Table 6.

**Table 6:** Sample 2 group level clustering indices.

***Construct***	***ICC(1)***	***ICC(2)***	***Median r_*wg*_(j)***
Civility climate	0.03	0.28	0.76
Accountability climate	0.04	0.37	0.67
Conflict resolution	0.01	0.12	0.62
Harassment responsiveness	0.06	0.45	0.39
CARES	0.06	0.43	0.77

*N* = 868.

### Construct validation

While the nature of this work was to some extent exploratory, we had some expectations about how the CARES would relate to other measures. For some of our hypotheses we expected strong positive correlations (>0.50), while for other hypotheses we expected weaker correlations (< 0.30). For one measure, we expected a weak negative correlation.

When individuals feel a sense of relationship and inclusion with the organization and with coworkers, those members will also experience an increased sense of integration with the organization (Blau, 1960). Following this logic, we expect that a positive interpersonal climate which encourages healthy conflict resolution, establishes respectful interactions as a workgroup norm, promotes fair treatment of all members, and discourages hostile and unhealthy behavior will, in turn, result in a feeling of integration in the workplace.

*Hypothesis 1: Interpersonal climate is strongly positively related (*>*0.50) to a feeling of integration in the workplace*.

Continuance commitment is the commitment felt by employees not as a result of an affective or normative desire to stay with an organization, but rather an intention to remain with an organization only because of a perceived lack of viable alternatives (Allen and Meyer, 1990; Meyer and Allen, 1984). Employees who have remained in an organization are likely to express a sense of continuance commitment in the absence of more salient drivers of a desire to remain with the organization. Negative work environments, such as those created by abusive supervision, are positively related to continuance commitment (Tepper, 2000). Climates which are exemplified by a positive sense of justice are negatively related to continuance commitment. We, therefore, expect a positive interpersonal climate exemplified by a sense of fair and respectful interactions will be negatively related to continuance commitment.

*Hypothesis 2: Interpersonal climate is weakly negatively (*<*-0.30) related to continuance commitment*.

Our expectation of a weak negative correlation between interpersonal climate and continuance commitment is based on our recognition that continuance commitment is driven by many factors extrinsic to the organizational climate and often specific to the personal constraints individual organizational members may be under due to circumstances beyond their current position.

Organizational citizenship behaviors (OCB's) are voluntary behaviors which are intended to benefit the organization and coworkers (Organ, 1988, 1997) and result from group norms (Ehrhart and Naumann, 2004). Therefore, workgroups who experience the group norm of a positive interpersonal climate—whose members are supportive and respectful of each other and do not have to guard against others unjustly taking credit for accomplishments, where affective and cognitive interpersonal ties are strengthened, and where members feel supported by both leaders and the institution—will include members who are more likely to engage in higher levels of organizational citizenship behaviors. Furthermore, individuals who operate within a positive interpersonal climate are expected to create positive affect and cognition directed toward coworkers and the organization and will engage in increased levels of OCB (Frazier et al., 2017; Organ and Konovsky, 1989). Such individuals will also develop a sense of social exchange and reciprocity with the foci of that climate (i.e., the institution, leaders, and workgroup members) leading to increased levels of citizenship behaviors (Settoon et al., 1996).

*Hypothesis 3: Interpersonal climate is strongly positively (*>*0.50) related to organizational citizenship behaviors*.

A positive interpersonal climate which includes expressions of interpersonal respect and civility and which guards against hostile, demeaning, and harassing behaviors at the levels of the institution, workgroup leadership, and coworker interactions will foster a climate where individuals are not excluded or harassed for exhibiting diverse aspects. Rather, it will foster a climate which is receptive to differences among its members resulting in increased levels of work unit diversity (WUD).

*Hypothesis 4: Interpersonal climate is strongly positively (*>*0.50) related to work unit diversity*.

Organizational climates have been shown to have strong and positive influences on corresponding individual behaviors within organizations (Schneider et al., 2013) which provides the basis for our expectations that climate is related to interpersonal experiences within an organization. Consequently, we expect that a positive interpersonal climate will be positively associated with workgroup member behavior-related experiences (WUE) exemplified by low levels of incivility and high levels of inclusion, cohesion, and citizenship.

*Hypothesis 5: Interpersonal climate is strongly positively (*>*0.50) related to work unit experiences*.

A positive interpersonal climate is an important component of creating a workgroup with an enduring level of professional integrity. The current study was conducted in research environments and, therefore, we here focus on research integrity. Research integrity is partially exemplified by fair and respectful leadership behaviors, respectful and supportive interpersonal interactions, and open and fair behaviors. As discussed above, these behaviors are fostered by a positive interpersonal climate emphasizing the importance of interpersonal respect and fairness, supportive leadership behaviors, and related institutional policies and practices.

*Hypothesis 6: Interpersonal climate is strongly positively (*>*0.50) related to research integrity climate*.

### Scale evaluation

#### Participants

We invited 376 members from Sample 1 who had completed a previous survey related to this research and consented to be invited to participate in this follow-up survey (Sample 1c in Figure 1). Participation was voluntary and participants were assured of the confidentiality of the study. Similar to previous samples, we omitted 33 responses due to suspect or missing responses, resulting in a usable sample of 343 completed surveys (a 91% participation rate). The demographic breakdown was as follows: 50% female, 45% male, and 5% not answering; 0.9% American Indian or Alaskan Native, 4% Asian, 1% Black or African American, 3% Hispanic or Latino, 0.3% Native Hawaiian or Pacific Islander, 69% white, 6% not listed or multiracial, and 16% not answering; 20% students, 26% early career researchers, 26% mid-career researchers, 25% late career researchers, and 4% not answering.

#### Measures

In addition to the workgroup climate measure (CARES), research integrity climate measure (SOURCE), integration item, and continuance commitment measure, the following listed constructs were collected in this survey. Responses to all measures were scaled from 1 (Not at All) to 5 (Completely) with an additional “No Basis for Judging” option.

#### Organizational citizenship behaviors (OCB)

Participants rated the helping behaviors of members of their workgroup using the 5-items helping scale from Ehrhart (2004). An example item is “Members of my work unit help others who have heavy workloads” (α = 0.94, ω = 0.95).

#### Work unit diversity climate (WUD)

Participants rated the diversity climate in their workgroup using the 4-item work unit diversity scale (McKay et al., 2008). An example item is “Leaders in my work unit demonstrate a visible commitment to diversity” (α = 0.92, ω = 0.95).

#### Work unit experiences (WUE)

Participants rated their individual experiences in their workgroup using the 13-item work unit environment scale developed by Vicki Magley (personal communication), and which includes items from the Cortina et al., Workplace Incivility Scale (Cortina et al., 2001), two items from the Martin and Hine measure of uncivil workplace behavior (Martin and Hine, 2005), and question items assessing cohesion, inclusion, and citizenship generated *de-novo* by Magley or modified by her from existing items to have an experiential/behavioral framing. An example item is “During the past year, were you ever in a situation in which any of your work unit members showed you genuine concern and courtesy” (α = 0.92, ω = 0.95).

We further assessed the validity of the CARES instrument by examining the relationship between it and measures of constructs at both the individual and work-unit levels which are expected to correlate with work-unit climate. The means, standard deviations, and correlations for these scales can be found in Table 7. Supporting Hypothesis 1, workgroup members' feelings of integration in the workplace correlated moderately to strongly positively with interpersonal climate as measured by the CARES instrument (*r* = 0.55) and its dimensions (*r* = 0.35 to 0.51). Supporting Hypothesis 2, continuance commitment correlated moderately negatively with interpersonal climate as measured by the CARES instrument (*r* = −0.20) and its dimensions (*r* = −0.11 to −0.23). Supporting Hypothesis 3, OCB correlated moderately to strongly positively with interpersonal climate as measured by the CARES instrument (*r* = 0.58) and its dimensions (*r* = 0.21 to 0.60). Supporting Hypothesis 4, WUD correlated moderately to strongly positively with interpersonal climate as measured by the CARES instrument (*r* = 0.72) and its dimensions (*r* = 0.35 to 0.76). Supporting Hypothesis 5, WUE correlated moderately to strongly positively with interpersonal climate as measured by the CARES instrument (*r* = 0.66) and its dimensions (*r* = 0.26 to 0.73). Finally, supporting Hypothesis 6, research integrity in the workgroup correlated moderately to strongly positively with interpersonal climate as measured by the CARES instrument (*r* = 0.78) and its dimensions (*r* = 0.56 to 0.79).

**Table 7:** Sample 2 means, standard deviations, and correlations among variables.

	**Mean**	**SD**	**1**	**2**	**3**	**4**	**5**
1. CARES	3.86	0.76					
2. Civility	4.12	0.75	0.84^***^				
3. Accountability	1.62	0.91	−0.74^***^	−0.61^***^			
4. Conflict	3.44	1.01	0.82^***^	0.71^***^	−0.45^***^		
5. Responsiveness	3.61	1.18	0.74^***^	0.42^***^	−0.30^***^	0.45^***^	
6. SOURCE	3.29	0.44	0.64^***^	0.61^***^	−0.18^***^	0.62^***^	0.61^***^

*N* = 868, ^*^p < 0.05, ^**^p < 0.01, ^***^p < 0.001. *P*-values < = 0.05 indicate that the observed/reported value in the table is statistically significantly different from zero applying the typical probability threshold of 0.05. *P*-values smaller than that indicate the same statistical significance, but at increasingly stringent probability thresholds.

## Discussion

Our goal in developing the CARES instrument is to provide leaders of organizations with a tool to support the effective promotion of high-quality work climates. It does so by providing organizational leaders the ability to collect reliable data to benchmark baseline conditions of working climates across organizational sub-units, identify well-functioning units, and identify those areas needing improvement. Such local and specific evidence can facilitate recognition of high-performing sub-units from which best practices can be gleaned and distributed throughout the organization. It can further inform and facilitate the development and deployment of appropriately targeted interventions for areas of concern. When leadership efforts to create organizational change are tailored to the specific concerns identified in local settings by organizational members, they are likely to be more effective than the more typical “blanket” policy edicts. The latter are typically and generically applied to all organizational members and units, are often perceived as unwanted bureaucratic compliance requirements, and thus regularly miss the mark, because they are not informed by the specific concerns of organizational members in specific organizational sub-units.

As a newly developed measure of interpersonal climates in organizations, the CARES instrument provides a novel tool that is useful to leaders in understanding dimensions of their organizational climates that are recognized as important to organizational and individual outcomes, productivity, wellbeing, and success. This detailed understanding of the climate at the level of the workgroup provides organizations with the ability to identify and respond to both exemplary and concerning administrative sub-units and to understand the climate within those units in a multi-dimensional manner.

As hypothesized, exploratory and confirmatory factor analyses identified multiple dimensions addressing unique but correlated aspects of interpersonal climates in organizations. Moreover, these four CARES dimensions of interpersonal climate correlate positively with important organizational outcomes such as workgroup integration, organizational citizenship behavior, work unit diversity climate, and work unit experiences, and negatively with important individual outcomes such as continuance commitment to one's employer. Further, we found moderate to strong correlations between work unit interpersonal climate and research integrity climate. Multi-group confirmatory factor analyses established the invariance of scale measures across a broad range of salient sub-groups. This speaks to questions of intersectionality, suggesting that different groups perceive and respond to the survey items using the same frames of reference, or cognitive schema.

Contributing to the strength and practicality of the CARES instrument is an extensive and broad-based initial literature review, our refinements of survey items to cover a broad spectrum of dimensions and features of interpersonal climates, our subsequent enlistment of subject matter experts to provide critical qualitative input that led to further item modifications, and our quantitative analyses with two independent samples for exploratory and confirmatory factor analyses. It is worth noting that, although the initial theorizing and development of the CARES instrument explicitly considered psychological safety as an important component to healthy interpersonal interactions, this aspect of interpersonal climate did not emerge from the data as a unique dimension. Instead, we observe that each of the dimensions which did emerge is likely to contribute in part to a perception of psychological safety and other beneficial proximal and distal climate outcomes among workgroup members. We encourage future research which examines additional outcomes of a comprehensive, interpersonal climate including perceptions of psychological safety.

We also view it as strength that our measures intentionally eschew the evaluation of organizational member attitudes and intentions, specifically because our theory of change does not require change in these factors for there to be changes in behavior, and in the organizational climate itself. It is our view that the most powerful and lasting source of organizational change is found in changing organizational climate which, in turn, shapes members attitudes, intentions, behaviors, and downstream outcomes such as individual and organizational health and performance. It is possible that changes in expectations of organizational members might itself lead to changes in attitudes, but it is not a requirement for there to be positive change. These strengths notwithstanding, there are also some limitations to this work that warrant mention.

Both financial and time limitations precluded our development and utilization of survey samples for this work that would have allowed us to cluster individual respondents within meaningful organizational sub-units (e.g., something like a two-stage, clustered sample, with individual respondents nested within work-units). This means that we have not been able here to assess the strength of climate measures in terms of perceptions of them being ^*^shared^*^ by respondents within and across organizational sub-units. This also precluded our ability to assess whether shared perceptions of climate were more strongly correlated with measured outcomes than individual perceptions of them. While we remain confident in the importance of the quality of interpersonal organizational climates as factors influencing important organizational and individual outcomes, it is worth noting that such measures of climate clearly do not capture all aspects of interpersonal behavior that may be of interest and concern. Specifically, such tools may not always illuminate the existence of “bad-apple” individuals within a work-unit, particularly if that individual's behavior is targeted on one or a small number of individuals and not perceived or observed by others in the work-unit and has not yet risen to the level where it negatively impacts work-unit climate. As noted recently by Dumitrescu (2022), “We do not know how someone behaves toward those less powerful than they unless we catch them unaware, or unless their targets trust us enough to tell us.” In addition to the bad-apple issue, it is likely that climate surveys may miss identifying work-units that are so highly toxic that their members simply do not feel safe speaking up about the issues in any context, no matter guarantees of anonymity, for fear of being identified and retaliated against. While we believe the CARES is valid and useful in a broad range of organizational types, our work here has been confined to research oriented organizational settings. Further work is needed to examine utility of the CARES in a wide range of organizational settings. And finally, although a 22-item survey is a notably brief instrument in order to robustly capture a multidimensional understanding of workgroup climate, it is prudent to recognize that respondent burden is an ever-present concern, especially in organization-wide surveys of members. As such, future work should consider the feasibility of even more abbreviated versions of the CARES scale, either retaining its dimensionality or focusing on capturing a unidimensional measure of workgroup climate consistent with this current instrument.

One potential mechanism through which organizational climate is thought to influence organizational member behavior is its influence on intrinsic motivation. Gagné and Deci (2005) theorize that humans have a trio of fundamental psychological needs: experiencing competence, autonomy, and relatedness. Aspects of social context, including organizational climates in work settings, can support or undermine meeting these basic needs, primarily through whether these organizational features support or undermine intrinsic motivation of organizational members (Gagné and Deci, 2005). Intrinsic motivation has been shown to correlate positively with many desired domains in workplaces, such as perceived organizational support, optimism, job satisfaction, wellbeing and self-reported physical health, while correlating negatively with turnover intentions, and psychological distress (Gagné et al., 2010). If organizational climates undermine members' feelings of competence, autonomy, or relatedness, this in turn can threaten the development and continuance of individual intrinsic motivation. These ideas have been most fully articulated in self-determination theory (Deci et al., 2017; Deci and Ryan, 2000).

Importantly, these theoretical expectations are supported by extensive evidence documenting associations between negative interpersonal experiences (e.g., incivility, sexual harassment) and both individual and organizational outcomes. Such outcomes may include decreased job satisfaction (Lim and Cortina, 2005), worker motivation, commitment, and organizational citizenship behaviors (Johnson and Indvik, 2001), increased work withdrawal and turnover intentions (Cortina et al., 2001; Griffin, 2010; Miner-Rubino and Reed, 2010), reduced psychological wellbeing (Cortina et al., 2001; Johnson and Indvik, 2001; Lim and Lee, 2011), counterproductive work behavior (Penney and Spector, 2005) and work performance (Caza and Cortina, 2007; Porath and Erez, 2009).

### Implications for use

The CARES assesses multiple dimensions of interpersonal climates in organizations, focused on civility, interpersonal accountability, harassment, and conflict resolution in organizational sub-units. Although intercorrelated (see Table 2), each of these dimensions is distinct, and it is inappropriate to use and treat the CARES as a unidimensional construct, nor is it developed or intended to be used as such. It is not meant to be a general-purpose sexual harassment survey, examples of which there are many, nor is it meant to serve as a DEI assessment tool, though there is some conceptual overlap with both of these types of instruments. In developing the CARES items, we also realized that some dimensions of concern in the interpersonal climates in organizations (e.g., sexual harassment responsiveness) are most frequently subject to policies and actions taken at organizational levels beyond the immediate work-unit, and that are, therefore, influenced by factors more properly seen as residing in the control of leaders of the larger institutional body. As such, we felt it important to retain items addressing these dimensions, even though they yield results that extend beyond the work-unit.

The survey tool is not an intervention *per se*, rather based on our experience with administering organizational climate surveys, we envision it as a means of surfacing issues of concern that may not be on the radar of organizational leaders yet warrant their attention proactively. Similarly, we envision it as a means of surfacing positive exemplars in an organization which should equally be brought to the attention of organizational leaders. We further expect that this instrument will be useful in assessing organizational change interventions, though its utility in this regard remains to be tested in future work. Our results here support the utility of the CARES survey across a broad range of disciplines, and types of organizations (including traditional academic research government, and industry settings).

## Conclusion

The importance of climates to individual and organizational outcomes has long been recognized. The CARES instrument answers a critical need to more richly understand multiple dimensions of organizational climate—civility, interpersonal accountability, conflict resolution, and institutional harassment responsiveness—and to discern these aspects of climate at a level of meaningful action—that of the workgroup. This instrument has been developed and demonstrated to be robust across a rich variety of organizational members, providing organizations and their leaders confidence in a consistent and actionable measure of the climate which exists in each of their work-units. These attributes suggest that the CARES survey offers both researchers and practitioners a powerful new tool in the work of understanding and improving organizational climates.

## Data Availability

The CARES instrument has been licensed through the Creative Commons 4.0 license, as follows: This work is licensed under the creative commons attribution-noncommercial-noderivatives 4.0 international license. To view a copy of this license, visit http://creativecommons.Org/licenses/by-nc-nd/4.0/ or send a letter to creative commons, PO box 1866, mountain view, CA 94042, USA. 
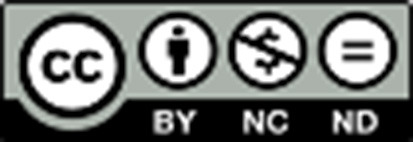 The raw data supporting the conclusions of this article will be made available by the authors, without undue reservation.

## Footnotes

^1^The results for the full AGU data set are not appreciably different than those for the second split half reported here and are available upon request from the corresponding author.

## Appendix 1

### CARES climate survey items

The prompt for the scale is “These questions are about the interpersonal climate of your primary work unit”. Response options include: 1 = “Not at All”, 2 = “Somewhat”, 3 = “Moderately”, 4 = “Very”, 5 = “Completely”. In addition, a “No Basis for Judging” response option is provided for all items.

**Civility Climate (work-unit level)**

1. How consistently do members of your work unit give appropriate credit for work effort and contributions?
2. How consistently do leaders in your work unit model respectful interpersonal behavior toward everyone, regardless of their identity or demographic characteristics?
3. Do members of your work unit value providing everyone equitable opportunities, regardless of their identity or demographic characteristics?
4. To what extent do leaders in your work unit create an environment where occasional errors are not a big deal?
5. To what extent are respectful interactions the norm in your work unit?
6. To what extent do leaders in your work unit create an environment that supports your personal and professional wellbeing?
7. To what extent are members of your work unit encouraging and supportive of each other?

**Interpersonal Accountability Climate**^2^ **(work-unit level)**

8. To what extent could your work unit be characterized as hostile and intimidating?
9. Do people in your work unit get away with hostile and intimidating behaviors?
10. Do members of your work unit use their power to intimidate, coerce, or demean others?
11. Do leaders in your work unit allow or create an environment that ignores rude or uncivil behavior?
12. Are top performers in your work unit allowed to get away with bad behavior?
13. To what extent are subtle slights, insults, or disrespectful comments tolerated in your work unit?
14. To what extent do performance pressures in your work unit lead to unhealthy interpersonal behavior?

**Conflict Resolution Climate (work-unit level)**

15. To what extent are leaders in your work unit helpful in resolving interpersonal disputes between coworkers, when they arise?
16. Do leaders in your work unit facilitate effective processes for handling disputes, either formally or informally?
17. How much are interpersonal behaviors considered as a component of performance reviews?

**Institutional Harassment Responsiveness (institution level)**

18. In cases of sexual harassment, does your institution hold everyone to the same standard regardless of who is being accused, who is reporting, or the nature of the complaint?
19. Does your institution take sexual harassment seriously?
20. How confident are you that your institution would hold leaders accountable who allowed sexual harassment?
21. Effective processes to report hostile and intimidating behavior should be well-defined, accessible, and safe to use. How effective are such processes at your institution?
22. Does your institution take visible actions to prevent sexual harassment (e.g., going beyond things like required trainings)?
