# Supplementary material for: Climate of Accountability, Respect, and Ethics Survey (CARES): development and validation of an organizational climate survey
Source: Front Res Metr Anal. 2025 Feb 25;10:1516726. doi: 10.3389/frma.2025.1516726 (PMC11894455; doi:10.3389/frma.2025.1516726)
Supplement: Supplementary file 1 [file Data_Sheet_1.pdf]

Author note: Some formatting was lost/corrupted when this file was initially output from Qualtrics and cannot be corrected. Content is otherwise as seen by invited survey respondents.

AGU ADVANCING EARTH AND SPACE SCIENCE | ncpre National Center for Professional & Research Ethics

Instructions

Interpersonal Work Climates and Research Integrity Development Study

You are being asked to participate in a voluntary research study. The purpose of this study is to extend a previously validated survey of organizational research climates. Participating in this study will involve only completion of this online survey. Your participation will last about 15 minutes. Risks related to this research do not include anything beyond those experienced in everyday life; benefits related to this research include contributing to efforts to improve our understanding of the ethical climate of research environments in order to improve the science done in those organizations. The alternative to participating in this study is to not participate.

Principal Investigator Name and Title: C. K. Gunsalus
Department and Institution: NCPRE, College of Engineering, University of Illinois Urbana-Champaign
Contact Information: gunsalus@illinois.edu
Sponsor: Sloan Foundation and the American Geophysical Union
Representative: Billy M. Williams, Senior Vice President, Ethics, Diversity & Inclusion, AGU

Why am I being asked?
You are being asked to be a participant in a research study about understanding the dynamics of organizational culture inside research intensive institutions. The purpose of this research is to be able to identify and articulate challenges in the culture for the purpose of improving them. You have been asked to participate in this research because you work in a research-focused job and can help us to understand the challenges faced in your work. Approximately 28,000 participants across the country will be invited to participate in this research.

Your participation in this research is voluntary. Your decision whether or not to participate will not affect your current or future dealings with the University of Illinois at Urbana-Champaign, nor with AGU. If you decide to participate, you are free to withdraw at any time without affecting those relationships.

Definitions

Definitions of terms used in the survey:

Equity: refers to fair and impartial treatment of people in your institution and work unit; not necessarily synonymous with equality.

Leaders: refers to those who hold positions of power or authority in your work unit or institution – either formally or informally.

Sexual misconduct: refers to any unwanted behavior of a sexual nature, and includes sexual or gender harassment (e.g. derogatory comments) and assault.

Work unit: Some of the questions in the survey ask about your primary work unit. Some people only work in one primary work unit while others have multiple. In answering the following questions, please think about the *one* work unit with which you spend most of your time.

Time-frame for responding: Given that since March 2020, the COVID-19 pandemic has severely curtailed many of the interpersonal interactions in peoples work-settings about which we are asking in this survey, we would like you to consider the period of time prior to these disruptions when answering the questions.

Are you directly involved in research in your current position at least 20% of your time? (Note, a response or “No” or “No basis for judging” will take you to the end of the survey.)

- Yes
- No
- No basis for judging

Adjectives

Your Work Unit:

Below is a list of adjectives that researchers might use to describe their work units. Please select up to five words from this list that you would use to describe the working climate in your **primary work unit**. Then “drop and drag” your selected words or phrases into the box to the right of this list. After you make your selections, please rank-order them within that box.

Some people only work in one primary work unit while others have multiple. In selecting these adjectives, please think about the *one* work unit with which you spend most of your time.

Abusive	Disorganized / Chaotic	Inefficient	Rigid / Inflexible
Anxiety provoking / Stressful	Disrespectful	Isolated / Isolating	Safe / Stable
Authoritarian	Dynamic	Just / Fair	Supportive
Careful	Dysfunctional	Lacking resources (e.g. funding)	Team / Group-oriented
Collaborative / Cooperative	Efficient	Leaderless	Time-Pressured / Rushed
Collegial	Encouraging	Learning / Discovery focused	Toxic
Communicative	Enthusiastic / Motivating	Low morale	Uncertain / Unpredictable
Competitive (productively)	Flexible	Micromanaging	Uncollaborative / Uncooperative
Competitive (unproductively)	Friendly	Non-communicative	Unfriendly
Creative/Curious	Fun	Open / Transparent	Unjust / Unfair
Dedicated	Hardworking	Overworked / Workaholic	Unproductive
Disciplined / Focused	Hostile	Productive	Unprofessional / Unethical
Discouraging	Inclusive / Open-minded	Professional / Ethical	Unsupportive
Discriminatory / Biased	Independent (empowering)	Relaxed	Welcoming
	Individualistic (not team-oriented)	Respectful	

Interpersonal Climate Work Unit - 36 Items

The next questions also ask about your **primary work unit** (we will ask about your institution as a whole in later questions). Some people only work in one primary work unit while others have multiple. In answering the following questions, please think about the *one* work unit with which you spend most of your time. We also recognize that you may have multiple leaders or only one leader in your work unit. We have used the plural form “leaders” throughout for simplicity.

Questions About the Interpersonal Climate of Your Primary Work Unit

	Not at All 1	Somewhat 2	Moderately 3	Very 4	Completely 5	No basis for judging
Are star performers in your work unit allowed to get away with bad behavior?						☐
Do leaders in your work unit allow or create an environment that ignores rude or uncivil behavior?						☐
To what extent do leaders of your work unit engage in hostile and intimidating behavior?						☐
To what extent do people feel safe speaking up about problems and tough issues in your work unit?						☐
To what extent do performance pressures in your work unit lead to unhealthy interpersonal behavior?						☐

	Not at All 1	Somewhat 2	Moderately 3	Very 4	Completely 5	No basis for judging	
How consistently do members of your work unit give appropriate credit for work effort and contributions?						☐	
Do leaders in your work unit take credit for the work of others?						☐	
To what extent are hostile and intimidating behaviors present in your work unit?						☐	
To what extent do leaders in your work unit create an environment where occasional errors are not a big deal?						☐	
How consistently do leaders in your work unit model respectful interpersonal behavior toward everyone, regardless of their identity or demographic characteristics?						☐	

	Not at All 1	Somewhat 2	Moderately 3	Very 4	Completely 5	No basis for judging
Do leaders in your work unit facilitate effective processes for handling disputes, either formally or informally?						☐
How confident are you that the privacy of all involved parties would be maintained if sexual misconduct were reported by someone in your work unit?						☐
How tolerated is behavior in your work unit that is disrespectful of others' space, time, or well-being (e.g. rude behavior)?						☐
To what extent are respectful interactions the norm in your work unit?						☐
How likely is it that a report of sexual harassment by a work unit member would be minimized or ignored?						☐

	Not at All	Somewhat	Moderately	Very	Completely	No basis for judging
	1	2	3	4	5	
To what extent do leaders in your work unit create an environment where sexual misconduct is unacceptable?						☐
Do members of your work unit abuse their power or authority (e.g. use their position to intimidate, coerce, or demean others)?						☐
Do members of your work unit value providing everyone equitable opportunities, regardless of their identity or demographic characteristics?						☐
To what extent do leaders in your work unit model respectful interactions no matter the situation (e.g., tight deadlines, short staffed)?						☐
How worried would you be that a member of your work unit would experience retaliation from the aggressor or others if they reported an instance of sexual						☐

	Not at All<br>1	Somewhat<br>2	Moderately<br>3	Very<br>4	Completely<br>5	No basis for judging	
How much pressure are members of your work unit under from leaders to behave dishonestly or unethically?						☐	
To what extent do leaders in your work unit create an environment that supports your personal and professional well-being?						☐	
To what extent are members of your work unit encouraging and supportive of each other?						☐	
How damaging to one's career would it be for someone in your work unit to raise concerns about sexual harassment?						☐	
To what extent are leaders in your work unit helpful in resolving interpersonal disputes between coworkers, when they arise?						☐	

	Not at All<br>1	Somewhat<br>2	Moderately<br>3	Very<br>4	Completely<br>5	No basis for judging	
To what extent could the environment of your work unit be characterized as hostile and intimidating?						☐	
How much are interpersonal behaviors considered as a component of performance reviews in your work unit?						☐	
Do you feel safe allowing yourself to be vulnerable with your work unit members?						☐	
If sexual misconduct occurred in your work unit, how confident are you that the responsible party would be held accountable, no matter who they were?						☐	
Do people in your work unit get away with hostile and intimidating behaviors?						☐	

	Not at All 1	Somewhat 2	Moderately 3	Very 4	Completely 5	No basis for judging	
How accepted is sexual misconduct among members of your work unit, either during or after hours?						☐	
To what extent are subtle slights, insults, or disrespectful comments tolerated in your work unit?						☐	
To what extent would there be negative consequences for a member of your work unit who behaved in a hostile and intimidating way?						☐	
Do leaders in your work unit treat all members equitably, regardless of their identity or demographic characteristics?						☐	
Would members in your work unit tolerate retaliation against a member who reported hostile and intimidating behavior?						☐	
Do members of your work unit undermine each other's efforts?						☐	

Research Integrity Climate Work Unit - 19 items

The next questions also ask about your **primary work unit** (we will ask about your institution as a whole in later questions). Some people only work in one primary work unit while others have multiple. In answering the following questions, please think about the *one* work unit with which you spend most of your time. We also recognize that you may have multiple leaders or only one leader in your work unit. We have used the plural form “leaders” throughout for simplicity.

Questions About the Research Integrity Climate of Your Primary Work Unit

	Not at All	Somewhat	Moderately	Very	Completely	No basis for judging	
	1	2	3	4	5		
How respectfully do advisors/supervisors treat advisees/supervisees?						☐	
How consistently do people in your work unit obtain permission or give due credit when using another's words or ideas?						☐	
How fair are your work unit's expectations with respect to publishing?						☐	
How fairly do advisors/supervisors treat advisees/supervisees?						☐	
How committed are people in your work unit to maintaining data integrity and data confidentiality?						☐	

	Not at All 1	Somewhat 2	Moderately 3	Very 4	Completely 5	No basis for judging	
How fair are your work unit's expectations of researchers/scholars for obtaining external funding?						☐	
How consistently do research/scholarly practices in your work unit follow established institutional policies?						☐	
How true is it that people in your work unit are more competitive with one another than they are cooperative?						☐	
How guarded are people in their communications with each other out of concern that someone else will "steal" their ideas.						☐	
How difficult is it to conduct research/scholarship in a responsible manner because of insufficient access to human resources such as statistical expertise, administrative or technical staff?						☐	

	Not at All 1	Somewhat 2	Moderately 3	Very 4	Completely 5	No basis for judging	
How true is it that pressure to obtain external funding has a negative effect on the integrity of research/scholarship in your work unit?						☐	
How consistently do administrators in your work unit (e.g., chairs, program heads) communicate high expectations for research/scholarly integrity?						☐	
How effectively are junior researchers socialized about responsible research practices?						☐	
How available are advisors/supervisors to their advisees/supervisees?						☐	
How committed are advisors in your work unit to talking with advisees about key principles of research integrity?						☐	

	Not at All<br>1	Somewhat<br>2	Moderately<br>3	Very<br>4	Completely<br>5	No basis for judging	
How difficult is it to conduct research/scholarship in a responsible manner because of insufficient access to material resources such as space, equipment, or technology?						☐	
How valued is honesty in proposing, performing, and reporting research/scholarship in your work unit?						☐	
How true is it that pressure to publish has a negative effect on the integrity of research/scholarship in your work unit?						☐	
How consistently do advisors/supervisors communicate to their advisees/supervisees clear performance expectations related to intellectual credit?						☐	

Interpersonal Climate Institution - 10 items

The next questions ask about your **institution as a whole**. In answering the

following questions, please think about the institution in which your primary work unit is located.

Questions About the Interpersonal Climate of Your Institution

	Not at All 1	Somewhat 2	Moderately 3	Very 4	Completely 5	No basis for judging	
Effective policies and procedures to prevent assaults or violence should be well-defined, accessible, and safe to use. How effective are such policies and procedures at your institution?						☐	
Do leaders at your institution take visible actions to prevent sexual harassment (e.g. going beyond things like required trainings)?						☐	
How confident are you that a sexual harassment complaint would be thoroughly investigated at your institution?						☐	
Effective processes to report sexual harassment should be well-defined, accessible, and safe to use. How effective are such processes at your institution?						☐	
How confident are you that your institution would hold leaders accountable who allowed sexual						☐	

	Not at All<br>1	Somewhat<br>2	Moderately<br>3	Very<br>4	Completely<br>5	No basis for judging	
How confident are you that you would know how to report an incident of sexual misconduct at your institution?						☐	
Does your institution take sexual harassment complaints seriously?						☐	
Effective processes to report hostile and intimidating behavior should be well-defined, accessible, and safe to use. How effective are such processes at your institution?						☐	
In cases of sexual harassment, does your institution hold everyone to the same standard regardless of who is being accused, who is reporting, or the nature of the complaint?						☐	
Do leaders at your institution take visible actions to prevent sexual assaults (e.g. going beyond things like required trainings)?						☐	

Research Integrity Climate Institution - 9 items

The next questions ask about your **institution as a whole**. In answering the following questions, please think about the institution in which your primary work unit is located.

Questions About the Research Integrity Climate of Your Institution

	Not at All<br>1	Somewhat<br>2	Moderately<br>3	Very<br>4	Completely<br>5	No basis for judging	
How committed are the senior administrators at your university (e.g., deans, chancellors, vice presidents) to supporting responsible research/scholarship?						☐	
How accessible are your university's policies/guidelines that relate to responsible research practices?						☐	
How effectively do the available educational opportunities at your university teach about responsible research practices (e.g., lectures, seminars, web-based courses)?						☐	
How confident are you that if you needed to report a case of suspected research misconduct, you would know where to turn to determine what procedures to follow?						☐	
How respectful to researchers are the regulatory committees or boards that review the type of						☐	

	Not at All 1	Somewhat 2	Moderately 3	Very 4	Completely 5	No basis for judging	
How accessible are individuals with appropriate expertise that you could ask for advice if you had a question about research ethics?						☐	
How fair to researchers/scholars are the regulatory committees or boards that review the type of research you do (e.g., IRB, IACUC)?						☐	
How effectively do the senior administrators at your university (e.g., deans, chancellors, vice presidents) communicate high expectations for research/scholarly integrity?						☐	
How well do the regulatory committees or boards that review your research/scholarship (e.g., IRB, IACUC) understand the kind of research you do?						☐	

Classification Items Workplace

Classification Items About your workplace

Which of the following best describes your place of work?

- Academic institution
- Research institution, public or state funded
- Research institution, private
- Industry
- ______ Other

To which AGU “Section” does your primary field of study belong?

______

Which of the following best describes your current status in your work unit?

______

How many years have you been affiliated with this work unit?

- < 1 year
- 1 - 5 years
- > 5 years

Is your work unit predominantly composed of people who are the same gender as you?

- Yes
- No

Is your work unit predominantly composed of people who are the same race or ethnicity as you?

- Yes
- No

	Not at all	Somewhat	Moderately	Very	Completely
How integrated within your department do you feel?	○	○	○	○	○

	Strongly disagree	Disagree	Neither agree nor disagree	Agree	Strongly agree
It would be very hard for me to leave my organization right now, even if I wanted to.	○	○	○	○	○
Too much in my life would be disrupted if I decided I wanted to leave my organization now.	○	○	○	○	○
I feel that I have too few options to consider leaving this organization.	○	○	○	○	○

Classification Items Participant

Classification Items About you

In which part of the world did you receive your Graduate training?

In what year did you obtain your highest degree?

What sex were you assigned at birth?

- Female
- Male
- Intersex
- Decline to answer

What is your current gender?

- Woman
- Man
- Transgender
- __________ A gender not listed
- Decline to answer

What is your ethnicity and race?

- American Indian / Alaska Native
- Asian
- Black or African American
- Hispanic or Latino
- Native Hawaiian or Other Pacific Islander
- White
- __________ Not listed
- Decline to answer

	Never	Hardly ever	Sometimes	Often	Always
How often do you find your work stressful?	○	○	○	○	○

	Not at all	Somewhat	Moderately	Very	Completely
How candidly did you respond to ALL of the questions above?	○	○	○	○	○

Do you have any comments or feedback to provide to us in regards to this survey?

Thanks

Thank you for taking the time to respond to our questions today.

If you are willing, we would like to ask your permission to contact you about a separate, but related, follow-up survey in a couple months. By agreeing to this contact now, you are NOT committing yourself to complete that survey, only to allowing us to re-contact you about it.

If you are willing to allow us to re-contact you, please provide your name and email address below:

Please click on the **Next → button** to submit your answers and end the survey.

First Name

Last Name

Email${q://QID209/ChoiceTextEntryValue}

Re-enter your email

Powered by Qualtrics
