# Supplementary material for: Climate of Accountability, Respect, and Ethics Survey (CARES): development and validation of an organizational climate survey
Source: Front Res Metr Anal. 2025 Feb 25;10:1516726. doi: 10.3389/frma.2025.1516726 (PMC11894455; doi:10.3389/frma.2025.1516726)
Supplement: Supplementary file 2 [file Supplementary_file_1.docx]

**SME Recruitment and Descriptives:**

The pool of SMEs recruited to this project were identified by the members of our team in part through our own professional networks, combined with the inclusion of graduate students, postdoctoral individuals, and faculty identified through lists from public entities including NASEM and NIH, and the labs of a private entity that we do not have permission to name, but which is a well-known and highly regarded research institution. We excluded one individual who was a Project Director at NASEM due to their indication that they felt they had a potential conflict of interest.

We purposefully included in the pool individuals across a range of academic standing, from graduate students through full professors as well as some editors of academic journals, e.g., Nature. In composing the pool, we had to make our own judgements about the sex of the candidate, and whether they were BIPOC or non-BIPOC.

The recruitment pool included three doctoral students, nine postdocs, three assistant professors, two associate professors, five full professors, and ten individuals for whom we did not have information on their current academic standing at time of recruitment, but were all known to be in academic positions.

The candidate pool included ten male-presenting and 24 female-presenting individuals, among whom we deemed twelve to be BIPOC and 23 to be non-BIPOC.

We did not solicit any demographic information from the eventual SME respondents, because we were interested in their content knowledge specifically, and we did not want to add any questions that might inhibit their honest and forthright responses to the item content we presented to them. Some of the respondents self-identified to us, in order to receive the $25 gift-certificate they were offered for their participation, and from that information we deemed three to be male, thirteen to be female, and two as unknown sex; two as BIPOC, fourteen as non-BIPOC, and two unknowns; two postdocs, one assistant professor, one associate professor, two full professors, two journal editors, and ten of unknown academic standing.

**Input Sought From SMEs:**

In our recruitment emails to the SME candidate pool, after we briefly described who we were as a team and the nature of our overall project, we said to them:

“Based on an extensive literature review of existing measures available in the public domain, our own thinking about these topics, and our team’s decades long experience in assessing academic research climates, we’ve developed a preliminary set of new question items to cover important ground pertaining to organizational research climates. We value your assessment of these items as a professional who may see things that we have missed, or perhaps just gotten somewhat wrong.

**How you can provide us your input:**

You can provide as much or as little input as you are willing. You can, of course, also skip any items you don’t wish to respond about. Beyond giving input on the existing items, if you see important topic areas that we’ve missed, we would value you pointing these out to us, so we can develop additional items to cover those areas. There is an open-ended response field at the end of the form in which you can provide such input.

By clicking on the following link, you will be directed to a Qualtrics data-collection form where we will ask you to provide your sense of the importance of including each item in the eventual survey, as well as telling us if you have other thoughts on how particular items might be modified to improve them.”

**Nature of Data Requested from SMEs:**

In addition to asking respondents to indicate how important they felt each question item was to include or exclude, we encouraged them to provide further insights about each item using open-ended text responses for each item. All respondents provided their ratings of the importance of either including or excluding each of the candidate question items, and nearly all respondents provided open-ended input suggesting revisions to multiple of the question items.

Once the responses had been obtained from the SMEs who elected to give us their input, our team met eleven times over a month-long period in Summer of 2020, spending nearly 100 person-hours reviewing the input received, classifying it by topic area (e.g. psychological safety, bullying, harassment, civility, etc.) as well as input discussing input received on mechanics (e.g. suggestions to provide definition of work-group, adding instructions of various sorts, and suggestions for added items to address content about which we had not asked). Where respondents convinced us that an item was problematic and should be excluded from our assessment, we dropped it. Where respondents suggested rewording, and persuaded us that such rewording of an item was necessary or would simply improve comprehension or clarity, we reworded items, in some cases ultimately developing completely different items than we had started with. Throughout this process we applied our own professional knowledge, skill, and many years of scale development experience to develop a set of items we felt were sufficiently comprehensive of interpersonal climates, and thus worth carrying forward for further testing in the large-scale survey fielding process that followed. We created the response scale regarding item “importance” specifically so SMEs could tell us if they felt a given item was centrally important to what we were trying to assess, or whether it might be in some way problematic, offensive, or misleading, in which case it would be very important to *not* include the item.

**SME Data Collection Template:**

We are asking you to evaluate potential question items for a new survey instrument to assess academic research climates with respect to psychological safety, civility, and the use, misuse, and abuse of power. This is not a survey about your research or working climate. We have identified you as having expertise or experience related to this topic and are asking for your help in evaluating the following items for inclusion in such an instrument.

Although **completely optional**, you are welcome to provide your name here, which will allow us to contact you personally if we have any follow-up questions about your feedback.

**Name**

Click here to enter text.

**Background**

The conceptual framework and background for this effort is grounded in organizational climate research. We define organizational climate as:

*The shared meaning organizational members attach to the events, policies, practices, and procedures they experience and the behaviors they see being rewarded, supported, and expected.*

Our focus here is on aspects of organizations that are easily observed and reported on by organizational members.

**Instructions**

In your roles as subject matter experts and *people in academia/labs,* we would like you to rate the following survey items designed to assess academic research climates with respect to professionalism, collegiality, and the use, misuse, and abuse of power. Please review each of the following 54 question items, and for each item indicate how important you think it would be to include for understanding these aspects of academic research climates. We also value your opinions and thoughts about whether the items need modification.

The final survey will use the following response options:

“Not at all,” “Somewhat,” “Moderately,” “Very,” and “Completely,” along with a “No basis for judging” option.

We have grouped these items under headings that provide a sense of the topic areas we are hoping to capture. Please feel free to skip over any topic area(s) where you feel you do not have adequate experience or knowledge to evaluate the question items.

Topic areas will be presented in the following order:

1. Psychological Safety - 13 items
2. Bullying - 9 items
3. Civility - 11 items
4. Harassment - 10 items
5. Assault - 11 item

**Please note**, at the end of this data collection form is a field where you can provide information on where we can send a $25 gift card as a token of our appreciation for your time and effort. This information will not be associated with the responses you are providing in this feedback survey.

**Section 1**

**Psychological Safety – 13 items**

The following items are intended to assess the psychological safety aspect of a research climate. For each of the items, indicate how important you think it would be to include that item for researchers to understand this aspect of a research climate. We also value your opinions and thoughts about whether each item might need modification.

At the end of this section, you will have the opportunity to provide additional thoughts not directly related to any specific item.

As a reminder, the final survey will use the following response options:
"Not at all", "Somewhat", "Moderately", "Very", and "Completely", along with a "No basis for judging" option.

| 1. **To what extent are people encouraging and supportive in your work unit?** | | | | |
| --- | --- | --- | --- | --- |
| Very important to include | Important to include | Some reservations | Important to exclude | Very important to exclude |
|  |  |  |  |  |
| **Do you have any thoughts, suggested modifications, or other comments you would like to share regarding item 1?**  Click here to enter text. | | | | |

| 1. **To what extent do leaders in your work unit create an environment that supports members’ well-being?** | | | | |
| --- | --- | --- | --- | --- |
| Very important to include | Important to include | Some reservations | Important to exclude | Very important to exclude |
|  |  |  |  |  |
| **Do you have any thoughts, suggested modifications, or other comments you would like to share regarding item 2?**  Click here to enter text. | | | | |

| 1. **To what extent is there a climate of trust in your work unit?** | | | | |
| --- | --- | --- | --- | --- |
| Very important to include | Important to include | Some reservations | Important to exclude | Very important to exclude |
|  |  |  |  |  |
| **Do you have any thoughts, suggested modifications, or other comments you would like to share regarding item 3?**  Click here to enter text. | | | | |

| 1. **To what extent are people in your work unit distrustful and suspicious of one another?** | | | | |
| --- | --- | --- | --- | --- |
| Very important to include | Important to include | Some reservations | Important to exclude | Very important to exclude |
|  |  |  |  |  |
| **Do you have any thoughts, suggested modifications, or other comments you would like to share regarding item 4?**  Click here to enter text. | | | | |

| 1. **How consistently do members of your work unit give credit where credit is due?** | | | | |
| --- | --- | --- | --- | --- |
| Very important to include | Important to include | Some reservations | Important to exclude | Very important to exclude |
|  |  |  |  |  |
| **Do you have any thoughts, suggested modifications, or other comments you would like to share regarding item 5?**  Click here to enter text. | | | | |

| 1. **Do leaders in your work unit treat members equitably, regardless of their identity or demographic characteristics?** | | | | |
| --- | --- | --- | --- | --- |
| Very important to include | Important to include | Some reservations | Important to exclude | Very important to exclude |
|  |  |  |  |  |
| **Do you have any thoughts, suggested modifications, or other comments you would like to share regarding item 6?**  Click here to enter text. | | | | |

| 1. **Do work unit members have equitable opportunities, regardless of their identity or demographic characteristics?** | | | | |
| --- | --- | --- | --- | --- |
| Very important to include | Important to include | Some reservations | Important to exclude | Very important to exclude |
|  |  |  |  |  |
| **Do you have any thoughts, suggested modifications, or other comments you would like to share regarding item 7?**  Click here to enter text. | | | | |

| 1. **To what extent are your work unit leaders trustworthy?** | | | | |
| --- | --- | --- | --- | --- |
| Very important to include | Important to include | Some reservations | Important to exclude | Very important to exclude |
|  |  |  |  |  |
| **Do you have any thoughts, suggested modifications, or other comments you would like to share regarding item 8?**  Click here to enter text. | | | | |

| 1. **To what extent could the environment of your work unit be characterized as hostile or toxic?** | | | | |
| --- | --- | --- | --- | --- |
| Very important to include | Important to include | Some reservations | Important to exclude | Very important to exclude |
|  |  |  |  |  |
| **Do you have any thoughts, suggested modifications, or other comments you would like to share regarding item 9?**  Click here to enter text. | | | | |

| 1. **To what extent do people feel safe speaking up about problems and tough issues in your work unit?** | | | | |
| --- | --- | --- | --- | --- |
| Very important to include | Important to include | Some reservations | Important to exclude | Very important to exclude |
|  |  |  |  |  |
| **Do you have any thoughts, suggested modifications, or other comments you would like to share regarding item 10?**  Click here to enter text. | | | | |

| 1. **To what extent do leaders in your work unit create an environment that destigmatizes occasional errors?** | | | | |
| --- | --- | --- | --- | --- |
| Very important to include | Important to include | Some reservations | Important to exclude | Very important to exclude |
|  |  |  |  |  |
| **Do you have any thoughts, suggested modifications, or other comments you would like to share regarding item 11?**  Click here to enter text. | | | | |

| 1. **How receptive are leaders in your work unit to hearing about job-related concerns?** | | | | |
| --- | --- | --- | --- | --- |
| Very important to include | Important to include | Some reservations | Important to exclude | Very important to exclude |
|  |  |  |  |  |
| **Do you have any thoughts, suggested modifications, or other comments you would like to share regarding item 12?**  Click here to enter text. | | | | |

| 1. **To what extent have you observed coworkers engaging in subtle slights, insults, or disrespect of someone that appeared to be based on their identity or demographic characteristics?** | | | | |
| --- | --- | --- | --- | --- |
| Very important to include | Important to include | Some reservations | Important to exclude | Very important to exclude |
|  |  |  |  |  |
| **Do you have any thoughts, suggested modifications, or other comments you would like to share regarding item 13?**  Click here to enter text. | | | | |

**Please feel free to provide any additional feedback you might have on capturing the psychological safety aspect of a research climate.**

Click here to enter text.

**Section 2**

**Bullying – 9 items**

The following items are intended to assess research climates with respect to bullying. For each of the items, indicate how important you think it would be to include that item for researchers to understand this aspect of a research climate. We also value your opinions and thoughts about whether each item might need modification.

At the end of this section, you will have the opportunity to provide additional thoughts not directly related to any specific item.

As a reminder, the final survey will use the following response options:
"Not at all", "Somewhat", "Moderately", "Very", and "Completely", along with a "No basis for judging" option.

| 1. **At your institution, are there effective reporting processes for when incidents of abusive, verbally aggressive, or threatening behavior occur?** | | | | |
| --- | --- | --- | --- | --- |
| Very important to include | Important to include | Some reservations | Important to exclude | Very important to exclude |
|  |  |  |  |  |
| **Do you have any thoughts, suggested modifications, or other comments you would like to share regarding item 1?**  Click here to enter text. | | | | |

| 1. **Do people in your work unit get away with abusive or aggressive behaviors in interacting with others?** | | | | |
| --- | --- | --- | --- | --- |
| Very important to include | Important to include | Some reservations | Important to exclude | Very important to exclude |
|  |  |  |  |  |
| **Do you have any thoughts, suggested modifications, or other comments you would like to share regarding item 2?**  Click here to enter text. | | | | |

| 1. **To what extent would there be reprisal if a member of your work unit reported behavior that was abusive, verbally aggressive, or threatening?** | | | | |
| --- | --- | --- | --- | --- |
| Very important to include | Important to include | Some reservations | Important to exclude | Very important to exclude |
|  |  |  |  |  |
| **Do you have any thoughts, suggested modifications, or other comments you would like to share regarding item 3?**  Click here to enter text. | | | | |

| 1. **To what extent are abusive, aggressive, or threatening behaviors present in your work unit?** | | | | |
| --- | --- | --- | --- | --- |
| Very important to include | Important to include | Some reservations | Important to exclude | Very important to exclude |
|  |  |  |  |  |
| **Do you have any thoughts, suggested modifications, or other comments you would like to share regarding item 4?**  Click here to enter text. | | | | |

| 1. **To what extent do leaders in your work unit engage in behavior that is abusive, verbally aggressive, or threatening?** | | | | |
| --- | --- | --- | --- | --- |
| Very important to include | Important to include | Some reservations | Important to exclude | Very important to exclude |
|  |  |  |  |  |
| **Do you have any thoughts, suggested modifications, or other comments you would like to share regarding item 5?**  Click here to enter text. | | | | |

| 1. **Do work unit members abuse their authority?** | | | | |
| --- | --- | --- | --- | --- |
| Very important to include | Important to include | Some reservations | Important to exclude | Very important to exclude |
|  |  |  |  |  |
| **Do you have any thoughts, suggested modifications, or other comments you would like to share regarding item 6?**  Click here to enter text. | | | | |

| 1. **To what extent would there be negative consequences for a work unit member who behaved in an abusive, aggressive or threatening manner?** | | | | |
| --- | --- | --- | --- | --- |
| Very important to include | Important to include | Some reservations | Important to exclude | Very important to exclude |
|  |  |  |  |  |
| **Do you have any thoughts, suggested modifications, or other comments you would like to share regarding item 7?**  Click here to enter text. | | | | |

| 1. **Are star performers in your work unit allowed to get away with bad behavior?** | | | | |
| --- | --- | --- | --- | --- |
| Very important to include | Important to include | Some reservations | Important to exclude | Very important to exclude |
|  |  |  |  |  |
| **Do you have any thoughts, suggested modifications, or other comments you would like to share regarding item 8?**  Click here to enter text. | | | | |

| 1. **Do leaders in your work unit take credit for the work of others?** | | | | |
| --- | --- | --- | --- | --- |
| Very important to include | Important to include | Some reservations | Important to exclude | Very important to exclude |
|  |  |  |  |  |
| **Do you have any thoughts, suggested modifications, or other comments you would like to share regarding item 9?**  Click here to enter text. | | | | |

**Please feel free to provide any additional feedback you might have on assessing research climates with respect to bullying.**

Click here to enter text.

**Section 3**

**Civility – 11 items**

The following items are intended to assess the civility aspect of research climates. For each of the items, indicate how important you think it would be to include that item for researchers to understand this aspect of a research climate. We also value your opinions and thoughts about whether each item might need modification.

At the end of this section, you will have the opportunity to provide additional thoughts not directly related to any specific item.

As a reminder, the final survey will use the following response options:
"Not at all", "Somewhat", "Moderately", "Very", and "Completely", along with a "No basis for judging" option.

| 1. **Is rude behavior displayed in your work unit?** | | | | |
| --- | --- | --- | --- | --- |
| Very important to include | Important to include | Some reservations | Important to exclude | Very important to exclude |
|  |  |  |  |  |
| **Do you have any thoughts, suggested modifications, or other comments you would like to share regarding item 1?**  Click here to enter text. | | | | |

| 1. **Do leaders in your work unit allow an environment that actively normalizes or passively ignores bad behavior?** | | | | |
| --- | --- | --- | --- | --- |
| Very important to include | Important to include | Some reservations | Important to exclude | Very important to exclude |
|  |  |  |  |  |
| **Do you have any thoughts, suggested modifications, or other comments you would like to share regarding item 2?**  Click here to enter text. | | | | |

| 1. **How consistently do leaders in your work unit model respectful behavior toward everyone?** | | | | |
| --- | --- | --- | --- | --- |
| Very important to include | Important to include | Some reservations | Important to exclude | Very important to exclude |
|  |  |  |  |  |
| **Do you have any thoughts, suggested modifications, or other comments you would like to share regarding item 3?**  Click here to enter text. | | | | |

| 1. **To what extent do leaders in your work unit help resolve disputes between coworkers, when they arise?** | | | | |
| --- | --- | --- | --- | --- |
| Very important to include | Important to include | Some reservations | Important to exclude | Very important to exclude |
|  |  |  |  |  |
| **Do you have any thoughts, suggested modifications, or other comments you would like to share regarding item 4?**  Click here to enter text. | | | | |

| 1. **To what extent do leaders in your work unit model respectful interactions no matter the situation (e.g., tight deadlines, short staffed)?** | | | | |
| --- | --- | --- | --- | --- |
| Very important to include | Important to include | Some reservations | Important to exclude | Very important to exclude |
|  |  |  |  |  |
| **Do you have any thoughts, suggested modifications, or other comments you would like to share regarding item 5?**  Click here to enter text. | | | | |

| 1. **How common is it that creativity is stifled in your work unit due to performance pressures?** | | | | |
| --- | --- | --- | --- | --- |
| Very important to include | Important to include | Some reservations | Important to exclude | Very important to exclude |
|  |  |  |  |  |
| **Do you have any thoughts, suggested modifications, or other comments you would like to share regarding item 6?**  Click here to enter text. | | | | |

| 1. **How consistently do leaders in your work unit model appropriate interpersonal behaviors?** | | | | |
| --- | --- | --- | --- | --- |
| Very important to include | Important to include | Some reservations | Important to exclude | Very important to exclude |
|  |  |  |  |  |
| **Do you have any thoughts, suggested modifications, or other comments you would like to share regarding item 7?**  Click here to enter text. | | | | |

| 1. **To what extent are respectful interactions the norm in your work unit?** | | | | |
| --- | --- | --- | --- | --- |
| Very important to include | Important to include | Some reservations | Important to exclude | Very important to exclude |
|  |  |  |  |  |
| **Do you have any thoughts, suggested modifications, or other comments you would like to share regarding item 8?**  Click here to enter text. | | | | |

| 1. **Do leaders in your work unit provide effective processes for handling disputes?** | | | | |
| --- | --- | --- | --- | --- |
| Very important to include | Important to include | Some reservations | Important to exclude | Very important to exclude |
|  |  |  |  |  |
| **Do you have any thoughts, suggested modifications, or other comments you would like to share regarding item 9?**  Click here to enter text. | | | | |

| 1. **To what extent do performance pressures in your work unit lead to unhealthy interpersonal behavior?** | | | | |
| --- | --- | --- | --- | --- |
| Very important to include | Important to include | Some reservations | Important to exclude | Very important to exclude |
|  |  |  |  |  |
| **Do you have any thoughts, suggested modifications, or other comments you would like to share regarding item 10?**  Click here to enter text.   \| 1. **Do people in your work unit undermine the efforts of others?** \| \| \| \| \| \| --- \| --- \| --- \| --- \| --- \| \| Very important to include \| Important to include \| Some reservations \| Important to exclude \| Very important to exclude \| \|  \|  \|  \|  \|  \| \| **Do you have any thoughts, suggested modifications, or other comments you would like to share regarding item 11?**  Click here to enter text. \| \| \| \| \| | | | | |

**Please feel free to provide any additional feedback you might have on civility aspects of research climates.**

Click here to enter text.

**Section 4**

**Harassment – 10 items**

The following items are intended to assess the aspects of harassment in research climates. For each of the items, indicate how important you think it would be to include that item for researchers to understand this aspect of a research climate. We also value your opinions and thoughts about whether each item might need modification.

At the end of this section, you will have the opportunity to provide additional thoughts not directly related to any specific item.

As a reminder, the final survey will use the following response options:
"Not at all", "Somewhat", "Moderately", "Very", and "Completely", along with a "No basis for judging" option.

| 1. **Does your institution take sexual harassment complaints seriously?** | | | | |
| --- | --- | --- | --- | --- |
| Very important to include | Important to include | Some reservations | Important to exclude | Very important to exclude |
|  |  |  |  |  |
| **Do you have any thoughts, suggested modifications, or other comments you would like to share regarding item 1?**  Click here to enter text. | | | | |

| 1. **In cases of sexual harassment, does your institution hold everyone to the same standard regardless of who is being accused, who is reporting, or the nature of the complaint?** | | | | |
| --- | --- | --- | --- | --- |
| Very important to include | Important to include | Some reservations | Important to exclude | Very important to exclude |
|  |  |  |  |  |
| **Do you have any thoughts, suggested modifications, or other comments you would like to share regarding item 2?**  Click here to enter text. | | | | |

| 1. **Do leaders at your institution take visible actions to prevent sexual harassment?** | | | | |
| --- | --- | --- | --- | --- |
| Very important to include | Important to include | Some reservations | Important to exclude | Very important to exclude |
|  |  |  |  |  |
| **Do you have any thoughts, suggested modifications, or other comments you would like to share regarding item 3?**  Click here to enter text. | | | | |

| 1. **How confident are you that a sexual harassment complaint would be thoroughly investigated at your institution?** | | | | |
| --- | --- | --- | --- | --- |
| Very important to include | Important to include | Some reservations | Important to exclude | Very important to exclude |
|  |  |  |  |  |
| **Do you have any thoughts, suggested modifications, or other comments you would like to share regarding item 4?**  Click here to enter text. | | | | |

| 1. **How confident are you that your institution would hold leaders accountable who allowed sexual harassment?** | | | | |
| --- | --- | --- | --- | --- |
| Very important to include | Important to include | Some reservations | Important to exclude | Very important to exclude |
|  |  |  |  |  |
| **Do you have any thoughts, suggested modifications, or other comments you would like to share regarding item 5?**  Click here to enter text. | | | | |

| 1. **To what extent does your institution have easy to follow procedures addressing sexual harassment?** | | | | |
| --- | --- | --- | --- | --- |
| Very important to include | Important to include | Some reservations | Important to exclude | Very important to exclude |
|  |  |  |  |  |
| **Do you have any thoughts, suggested modifications, or other comments you would like to share regarding item 6?**  Click here to enter text. | | | | |

| 1. **How damaging to one’s career would it be to raise concerns about sexual harassment?** | | | | |
| --- | --- | --- | --- | --- |
| Very important to include | Important to include | Some reservations | Important to exclude | Very important to exclude |
|  |  |  |  |  |
| **Do you have any thoughts, suggested modifications, or other comments you would like to share regarding item 7?**  Click here to enter text. | | | | |

| 1. **How likely is it that a report of sexual harassment by a work unit member would be minimized or ignored?** | | | | |
| --- | --- | --- | --- | --- |
| Very important to include | Important to include | Some reservations | Important to exclude | Very important to exclude |
|  |  |  |  |  |
| **Do you have any thoughts, suggested modifications, or other comments you would like to share regarding item 8?**  Click here to enter text. | | | | |

| 1. **Do offensive or inappropriate sexual jokes, or comments occur in your work unit?** | | | | |
| --- | --- | --- | --- | --- |
| Very important to include | Important to include | Some reservations | Important to exclude | Very important to exclude |
|  |  |  |  |  |
| **Do you have any thoughts, suggested modifications, or other comments you would like to share regarding item 9?**  Click here to enter text. | | | | |

| 1. **To what extent is sexist behavior a problem in your work unit?** | | | | |
| --- | --- | --- | --- | --- |
| Very important to include | Important to include | Some reservations | Important to exclude | Very important to exclude |
|  |  |  |  |  |
| **Do you have any thoughts, suggested modifications, or other comments you would like to share regarding item 10?**  Click here to enter text. | | | | |

**Please feel free to provide any additional feedback you might have on aspects of harassment in research climates.**

Click here to enter text.

**Section 5**

**Assault/Sexual Misconduct – 11 items**

The following items are intended to assess the aspects of assault and sexual misconduct in research climates. For each of the items, indicate how important you think it would be to include that item for researchers to understand this aspect of a research climate. We also value your opinions and thoughts about whether each item might need modification.

At the end of this section, you will have the opportunity to provide additional thoughts not directly related to any specific item.

As a reminder, the final survey will use the following response options:
"Not at all", "Somewhat", "Moderately", "Very", and "Completely", along with a "No basis for judging" option.

| 1. **To what extent does your institution provide effective assault/violence prevention policies and procedures?** | | | | |
| --- | --- | --- | --- | --- |
| Very important to include | Important to include | Some reservations | Important to exclude | Very important to exclude |
|  |  |  |  |  |
| **Do you have any thoughts, suggested modifications, or other comments you would like to share regarding item 1?**  Click here to enter text. | | | | |

| 1. **How effective is the sexual misconduct training at your institution? (If there is no formal training, please indicate NBFJ)?** | | | | |
| --- | --- | --- | --- | --- |
| Very important to include | Important to include | Some reservations | Important to exclude | Very important to exclude |
|  |  |  |  |  |
| **Do you have any thoughts, suggested modifications, or other comments you would like to share regarding item 2?**  Click here to enter text. | | | | |

| 1. **To what extent do leaders at your institution take visible actions to prevent sexual assault?** | | | | |
| --- | --- | --- | --- | --- |
| Very important to include | Important to include | Some reservations | Important to exclude | Very important to exclude |
|  |  |  |  |  |
| **Do you have any thoughts, suggested modifications, or other comments you would like to share regarding item 3?**  Click here to enter text. | | | | |

| 1. **To what extent are there effective services at your institution for people who experienced sexual assault?** | | | | |
| --- | --- | --- | --- | --- |
| Very important to include | Important to include | Some reservations | Important to exclude | Very important to exclude |
|  |  |  |  |  |
| **Do you have any thoughts, suggested modifications, or other comments you would like to share regarding item 4?**  Click here to enter text. | | | | |

| 1. **How confident are you that you would know how to report an incident of sexual misconduct at your institution?** | | | | |
| --- | --- | --- | --- | --- |
| Very important to include | Important to include | Some reservations | Important to exclude | Very important to exclude |
|  |  |  |  |  |
| **Do you have any thoughts, suggested modifications, or other comments you would like to share regarding item 5?**  Click here to enter text. | | | | |

| 1. **How confident are you that members of your work unit would know how to get help if someone experienced sexual misconduct?** | | | | |
| --- | --- | --- | --- | --- |
| Very important to include | Important to include | Some reservations | Important to exclude | Very important to exclude |
|  |  |  |  |  |
| **Do you have any thoughts, suggested modifications, or other comments you would like to share regarding item 6?**  Click here to enter text. | | | | |

| 1. **To what extent is sexual misconduct a problem in your work unit?** | | | | |
| --- | --- | --- | --- | --- |
| Very important to include | Important to include | Some reservations | Important to exclude | Very important to exclude |
|  |  |  |  |  |
| **Do you have any thoughts, suggested modifications, or other comments you would like to share regarding item 7?**  Click here to enter text. | | | | |

| 1. **How worried would you be about there being reprisals if someone in your work unit reported an instance of sexual assault?** | | | | |
| --- | --- | --- | --- | --- |
| Very important to include | Important to include | Some reservations | Important to exclude | Very important to exclude |
|  |  |  |  |  |
| **Do you have any thoughts, suggested modifications, or other comments you would like to share regarding item 8?**  Click here to enter text. | | | | |

| 1. **How confident are you that privacy would be maintained if a member of your work unit made a sexual misconduct report?** | | | | |
| --- | --- | --- | --- | --- |
| Very important to include | Important to include | Some reservations | Important to exclude | Very important to exclude |
|  |  |  |  |  |
| **Do you have any thoughts, suggested modifications, or other comments you would like to share regarding item 9?**  Click here to enter text. | | | | |

| 1. **If sexual misconduct occurred in your work unit, how confident are you that the responsible party would be held accountable, no matter who that was?** | | | | |
| --- | --- | --- | --- | --- |
| Very important to include | Important to include | Some reservations | Important to exclude | Very important to exclude |
|  |  |  |  |  |
| **Do you have any thoughts, suggested modifications, or other comments you would like to share regarding item 10?**  Click here to enter text. | | | | |

| 1. **To what extent do leaders in your work unit create an environment where sexual misconduct is unacceptable?** | | | | |
| --- | --- | --- | --- | --- |
| Very important to include | Important to include | Some reservations | Important to exclude | Very important to exclude |
|  |  |  |  |  |
| **Do you have any thoughts, suggested modifications, or other comments you would like to share regarding item 11?**  Click here to enter text. | | | | |

**Please feel free to provide any additional feedback you might have on aspects of assault and sexual misconduct in research climates.**

Click here to enter text.
