# Supplementary material for: Climate of Accountability, Respect, and Ethics Survey (CARES): development and validation of an organizational climate survey
Source: Front Res Metr Anal. 2025 Feb 25;10:1516726. doi: 10.3389/frma.2025.1516726 (PMC11894455; doi:10.3389/frma.2025.1516726)
Supplement: Supplementary file 3 [file Supplementary_file_2.docx]

Among our supplemental materials we have included an Excel workbook containing artifacts of our team’s process of moving from the initial literature search identification of many hundreds of candidate question items down to the roughly n=130 items that we moved forward with in the subsequent development of the CARES instrument.

This workbook reflects only one part of the overall item development process, but we feel it illuminates the essential nature of our item selection and development process. The complexity of the workbook warrants some brief guidance in parsing it, as the document was never intended to be published, but rather, was a compendium for our own team to use as we moved through a process that entailed literally thousands of decision points.

The reader should start with the fourth tab in the workbook, titled “Cells” which provides a grid of the dimensions of organizational climate we sought to tap, as well as the content domains. The links in this grid allow one to navigate to particular content areas to see what candidate items fell into a particular cell of this grid. For instance, clicking on the “3B” link in cell C4, takes one to the 3B worksheet, which contains 62 candidate question items that we considered for inclusion related to leadership practices with respect to harassment. Of the cells defined by our two-dimensional grid, there were two only two cells where we did not identify any candidate items. Specifically, cells B5 and B7. In addition to the raw question items pulled from the literature, each worksheet includes various indexing columns, citation information, response categories, instructions, and our own notes. We discovered early on in our search process that many extant question items tapped more than one of our dimensions of interest, so the reader will note columns labeled “Dim 1” and “Dim 2” where we tracked that information.

It is worth noting that the dimensions with which we started our development process are somewhat different than those that eventually arose as parts of the CARES instrument. This is because throughout our scale development process, we let the data we collected, whether from the SMEs or from our large-scale surveys, and the analyses thereof, guide our decision-making. This is a hallmark of good research.

In addition to the worksheets specific to grid cells, there are some summary worksheets as well. The most pertinent of these is the first worksheet in the workbook, titled “Keepers” which contains the subset of items we ultimately retained from the literature review process.
